# Cerebellar complex spikes multiplex complementary behavioral information

**DOI:** 10.1371/journal.pbio.3001400

**Published:** 2021-09-16

**Authors:** Akshay Markanday, Junya Inoue, Peter W. Dicke, Peter Thier

**Affiliations:** 1 Hertie Institute for Clinical Brain Research, Tübingen, Germany; 2 Graduate School of Neural and Behavioral Sciences, International Max Planck Research School, Tübingen University, Tübingen, Germany; 3 Werner Reichardt Centre for Integrative Neuroscience, Tübingen University, Tübingen, Germany; Ecole Polytechnique Federale de Lausanne, SWITZERLAND

## Abstract

Purkinje cell (PC) discharge, the only output of cerebellar cortex, involves 2 types of action potentials, high-frequency simple spikes (SSs) and low-frequency complex spikes (CSs). While there is consensus that SSs convey information needed to optimize movement kinematics, the function of CSs, determined by the PC’s climbing fiber input, remains controversial. While initially thought to be specialized in reporting information on motor error for the subsequent amendment of behavior, CSs seem to contribute to other aspects of motor behavior as well. When faced with the bewildering diversity of findings and views unraveled by highly specific tasks, one may wonder if there is just one true function with all the other attributions wrong? Or is the diversity of findings a reflection of distinct pools of PCs, each processing specific streams of information conveyed by climbing fibers? With these questions in mind, we recorded CSs from the monkey oculomotor vermis deploying a repetitive saccade task that entailed sizable motor errors as well as small amplitude saccades, correcting them. We demonstrate that, in addition to carrying error-related information, CSs carry information on the metrics of both primary and small corrective saccades in a time-specific manner, with changes in CS firing probability coupled with changes in CS duration. Furthermore, we also found CS activity that seemed to predict the upcoming events. Hence PCs receive a multiplexed climbing fiber input that merges complementary streams of information on the behavior, separable by the recipient PC because they are staggered in time.

## Abstract 2

Purkinje cell (PC) discharge, the only output of cerebellar cortex, involves both high-frequency simple spikes and low-frequency complex spikes; the function of the latter, determined by a PC’s climbing fibre input, remains controversial. This study shows that PCs receive a multiplexed climbing fibre input that merges complementary streams of information relevant for behaviour.

## Introduction

The cerebellocortical Purkinje cell (PC) is unique in that it fires 2 types of action potentials, high-frequency simple spikes (SSs) and low-frequency complex spikes (CSs). SSs, which represent the integrated influence of information mediated via the mossy fiber-parallel fiber system and modulatory influences of interneurons, are fired at frequencies high enough to have an impact on target neurons outside the cerebellar cortex. They are thought to be the sole carrier of information allowing extracerebellar structures to optimize movement kinematics. CSs, on the other hand, are local manifestations of the second stream of input information conveyed by climbing fibers impinging onto target PCs. The strong excitatory influence of these CSs is thought to drive long-term plasticity at parallel fiber-to-PC synapses, which forms the basis of the first and arguably the most influential concept on the role of the climbing fiber input in reporting motor errors [1–3]. Within the framework of this Marr–Albus–Ito (MAI) concept, information on motor errors, conveyed via CSs, is used to drive motor learning by adjusting the PCs’ SS output to implement behavioral amendments necessary for avoiding the future reoccurrence of an error.

While several studies have been able to lend support to the MAI concept [4–14], others have unveiled features, not to be expected within this classical framework. For example, based on the clock-like regularity and precision of the firing of inferior olive neurons (the source of climbing fibers) Llinás and colleagues suggested a role of CSs in the temporal segmentation of movements [15–17]. Since then, many more observations on CS signaling have been made that require adaptations of the classical concept or even alternatives to it. For instance, studies on oculomotor adaptation in monkeys have suggested that CS discharge may not only initiate learning but, moreover, help to stabilize learning by reverberating information on past errors [18–20]. Fully compatible with this idea, also, experiments on eyeblink conditioning in mice have demonstrated that the same CSs that initially encode unexpected errors (air-puffs) also predict future errors by responding to a conditioned stimulus of a different sensory modality (LED or tone) reflecting information on past errors that serves as proxy of future errors [21]. Support for the role of CSs in encoding movement kinematics in addition to representing position errors comes from manual tracking experiments on monkeys [22]. Finally, more recently, yet another nonclassical role of CSs in conveying information on reward signals in a reactive and predictive manner has been put forward by studies involving visuomotor tracking experiments in mice [23] and smooth-pursuit eye movements in monkeys [24]. Additionally, it has also been shown that not only the CS firing probability but also its duration might be informative [25]. Yang and Lisberger [26–27] have argued that CS duration may indeed mediate error information instructing smooth-pursuit adaptation and suggested that instructive signals may adapt the spikelet architecture in a graded manner during motor learning.

It is a remarkable irony that CSs, notorious for extremely low discharge rates [28–33], hardly suitable to carry rich information, should accommodate such a diversity of behaviorally relevant signals. This, therefore, begs the question whether there is just one true function of CS with all the other attributions wrong, or are these different findings just a part of a larger puzzle? Could it be that we, cerebellar physiologists, share the fate of the blind monks in the ancient Indian parable, examining different parts of an elephant, each mistaking the respective part felt for the whole elephant? Alternatively, can the diversity of results, collected from different studies, be reconciled within a single task that may suggest that distinct pools of PCs might be recipients of specific streams of information conveyed by the climbing fibers? To answer these questions, we recorded CSs from the monkey oculomotor vermis (OMV) during a simple to and fro saccade task. By exploiting the natural variability in saccade endpoints that often evoked corrective saccades, we demonstrate that the same CS units that carry information on endpoint errors are also informative on primary and corrective saccade kinematics. We found that this information is represented not only by CS firing rates but also by CS duration. Additionally, we also found that a significant portion of CS units, which responded with a certain latency to trial onset, may predict the upcoming movement. We argue that probably every PC receives a multiplexed climbing fiber input that merges different and complementary streams of information on the behavior at stake, potentially separable by the recipient PC because they are staggered in time.

## Materials and methods

### Animals, preparation, surgical procedures, and recording methods

All neural and behavioral data used in this study were collected from 2 adult male rhesus macaques (*Macaca mulatta*), monkey K (age: 10 years) and monkey E (age: 8 years) that were purchased from the German Primate Center, Göttingen. All training, experiments, and surgical procedures abided by the rules set by German and European law as well as the National Institutes of Health’s *Guide for the Care and Use of Laboratory Animals* and were approved by the local authority (Regierungspräsidium Tübingen) for animal care under veterinary licenses N7/18 and N4/14. All procedures were carefully supervised by the veterinary service of Tübingen University.

Animals were trained to voluntarily enter an individually customized primate chair and get accustomed to the setup environment. After successful chair training, which could last for up to 3 months, the first major surgical procedure was conducted. During this procedure, titanium foundations of all implants were attached to the skull with titanium bone screws and allowed to rest under the subsequently reclosed skin for a period of approximately 3 to 4 months to ensure the long-term stability of the implant foundations. For the commencement of head fixation and experimental training, a titanium-based hexagonal tube-shaped head post was fixed to the base of the implanted head holder via the locally opened skin that allowed us to painlessly immobilize the head during experiments. At the same time, we implanted magnetic scleral search coils [34,35] to record eye position with high precision. After 2 to 3 weeks of recovery from the surgical procedures, monkeys were trained further on the tasks at stake. Once proficient, we opened the skin above the already implanted chamber foundation in order to attach the upper part of the cylindrical titanium recording chamber, tilting backward by an angle of 30° with respect to the frontal plane, right above the midline of the cerebellum and trepanated the skull within the confines of the chamber. The position and orientation of the chamber had been carefully planned based on presurgical MRI and was later confirmed by postsurgical MRI. This allowed reliable electrode access to our region of interest, the OMV (lobules VIc/VIIa). All surgical procedures were performed under aseptic conditions using general anesthesia (see Arnstein and colleagues [36] for more details). All vital physiological parameters (blood pressure, body temperature, heart rate, pO_2_, and pCO_2_) were closely monitored. After surgery, analgesics (buprenorphine) were delivered to ensure painless recovery. Regular ethograms were recorded to keep track of monkeys’ progress until full recovery.

### Behavioral task

**Fig 1:**
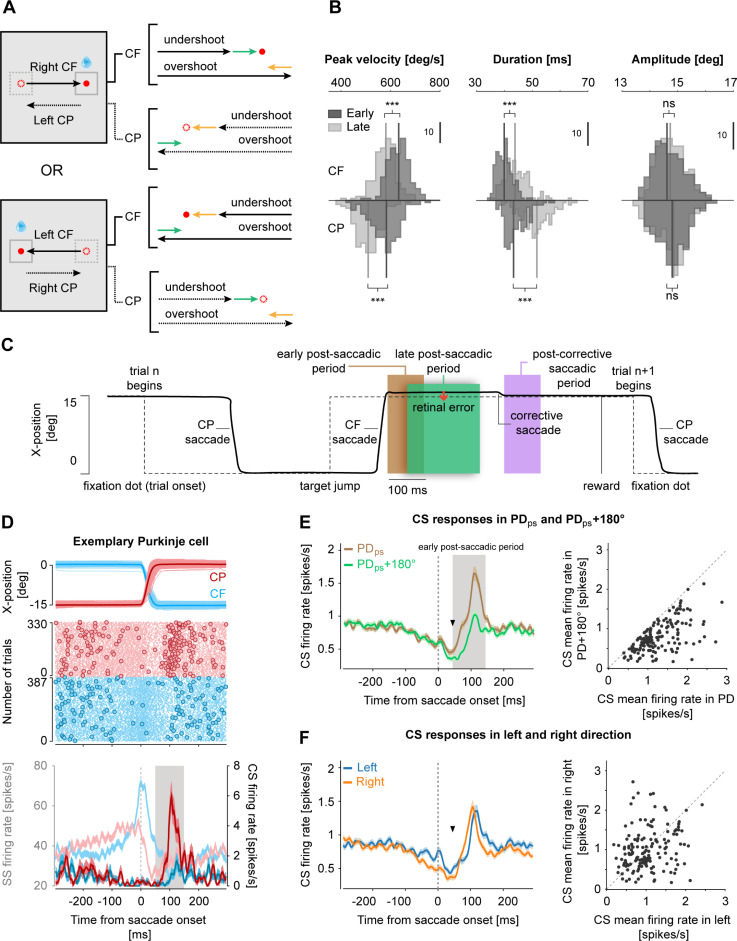
The influence of repetitive saccade paradigm on saccade behavior and encoding of primary saccade direction by CSs. **(A)** Experimental paradigm showing 2 separate sessions in which the rewarded CF saccades were made either toward the right (top panel) or left (bottom panel). And, in turn, the resulting CP saccades were made in the opposite direction. As shown in the illustrations, within a session, both CF (solid arrows) and CP (dashed arrows) saccades could either over- or undershoot the target location resulting in leftward (yellow arrows) and rightward errors (green arrows), respectively. Therefore, while saccades made in the same direction caused errors in opposite directions, saccades in opposite directions, for example, overshooting CF saccade to the right and undershooting CP saccade to the left could lead to errors in the same direction (i.e., leftward errors). **(B)** Histograms comparing peak velocity (left), duration (middle), and amplitude (right) of early (dark gray) and late (light gray) 30 trials of CF and CP saccades, pooled across 160 sessions. Solid vertical lines represent the median values. Vertical scale bars represent the number of sessions. **(C)** An illustration showing the temporal order of different events relative to saccade behavior (solid black trace) within a trial and the temporal relationship of various analytical periods, i.e., *“early postsaccadic period”* (brown shaded region; 0–100 ms from primary saccade offset), *“late postsaccadic period”* (green shaded region; 50–250 ms from primary saccade offset), and *“postcorrective saccadic period”* (purple shaded region; 0–100 ms from corrective saccade offset), relative to saccade behavior. Dashed lines indicate the position of the target dot at any given point in time. Note the overlap between the early and late postsaccadic period. **(D)** Response of an exemplary PC to left CF (blue) and right CP (red) saccades (upper panel). Raster plot (middle panel) of SSs (dots) and CSs (circles) and their corresponding mean (±SEM) firing rates (bottom panel) are shown in faded and bright colors, respectively. Note, how the probability of CS occurrence increases during the *“early postsaccadic period”* (gray shaded region, 0–100 ms from saccade end) for the rightward but not leftward saccades, suggesting that the PC’s preferred direction for primary saccades (PD_*ps*_) pointed in the rightward direction. Also note, both CF and CP saccades were followed by errors in both directions (see variability in individual saccade trajectories). All data are aligned to saccade onset (dashed gray line). **(E, F)** CS population response (mean ± SEM; *N =* 151 PCs) in PC’s preferred (PD_*ps*_) and antipreferred (PD_*ps*_+180°) direction (regardless of left, right, CF, and CP) of primary saccades (E, left panel) reveals a strong difference during the *“early postsaccadic period”* (gray shaded region), as compared to CS responses for primary saccades made in the left and right direction (F, left panel), regardless of CF and CP saccades. Inverted black triangles in E and F (left panels) represent the average saccade offset. All data are aligned to saccade onset (dashed gray line). Panels to the right in E and F show scatter plots highlighting individual differences between the PD_*ps*_ and PD_*ps*_+180° and left and right directions. Each dot represents the mean of CS firing rate calculated during the *“early postsaccadic period”* for each PC. Gray dashed lines are the unity lines. Underlying data available from the Dryad Digital Repository: [https://doi.org/10.5061/dryad.d51c5b03m]. CF, centrifugal; CP, centripetal; CS, complex spike; PC, Purkinje cell; SS, simple spike.

We trained the 2 monkeys on a fatigue-inducing repetitive fast eye movements (saccades) task (Fig 1A). A trial was initiated whenever the monkeys moved their eye gaze into an invisible fixation window (2 × 2 deg) centered on a red fixation dot of diameter, 0.2 deg that was displayed at the center of a CRT monitor, placed at a distance of 38 cm in front of the subject. After a short fixation period varying from 400 to 600 ms relative to trial onset, the fixation dot disappeared and at the same time, a target appeared (go-signal) either on the left or—in other sessions—on the right at 15 deg eccentricity with features, matching to that of the fixation dot. In response, the monkey made a fast eye movement (saccade) toward the new target location, which was considered correct only if the eye gaze landed within an invisible fixation window (2 × 2 deg) centered on the target. Every center-out (= centrifugal (CF)) saccade (Fig 1A, solid arrows) made correctly toward the target was rewarded with an instantaneously delivered drop of water. Approximately 700 to 900 ms after the go-signal, the peripheral target disappeared, and the central fixation dot reappeared. In order to proceed with the next CF trial, the monkeys readily executed short-latency back saccade from the peripheral target location to the fixation dot—centripetal (CP) saccades (dashed arrows)—although these CP saccades were not rewarded. As shown in Fig 1B (and S1 Fig), the metric and kinematic structure of CP and CF saccades were very similar, their opposite directions notwithstanding, and both exhibited saccadic fatigue [37], characterized by a gradual drop in peak velocity, compensated by an increase of duration, keeping saccade amplitude stable. Although initially designed to induce a gradual decline in saccade velocities, another important aspect of the paradigm was that it allowed us to systematically tease apart the influences of postsaccadic errors from those of primary saccade kinematics on CS firing. This was possible because of the natural variability in primary saccade endpoints scattered around the target location (S1C and S1D Fig), which resulted in both over- and undershoots, often followed by corrective secondary saccades. The sequence of events relative to saccade behavior within a trial is conveyed by a schematic illustration in Fig 1C. The large number of trials that varied per session (median: 326 trials) depending on the motivation of the monkey to perform the task as well as on the time for which a PC could be kept well isolated further allowed us to reliably measure subtle changes in CS firing rates as well as changes in CS duration without resorting to any sophisticated statistical approaches. The duration of each trial was 1,200 ms. A Linux-based in-house software, NREC (http://nrec.neurologie.uni-tuebingen.de), was used to control the experiment and to collect and preprocess data.

### Electrophysiological recordings of Purkinje cells in the oculomotor vermis

We performed extracellular recordings from PCs using glass-coated tungsten microelectrodes (impedance: 1 to 2 MΩ) that were purchased from Alpha Omega Engineering, Nazareth, Israel. The position of the electrodes, which were targeted toward the OMV, was controlled using a modular multielectrode manipulator (Electrode Positioning System and Multi-Channel Processor, Alpha Omega Engineering). The identity and the exact coordinates of the OMV predicted by the MRI scans were confirmed by physiological criteria, i.e., the presence of a dense saccade-related background activity, reflecting multiunit granule cells activity. To differentiate action potentials from the underlying LFP signals, extracellular potentials, recorded at the sampling rate of 25 KHz, were high band-pass filtered (300 Hz to 3 KHz) and low-pass filtered (30 Hz to 400 Hz), respectively. A total of 160 PCs were recorded out of which 151 were considered for analysis. A table summarizing the total number of PCs recorded from each monkey, either in left, right, or both directions, can be found in S1 Table.

### Identification and detection of simple spikes and complex spikes

The final identification of individual PC units relied on the demonstration of the 2 types of actions potentials they fire, the high-frequency SS discharge and the low-frequency CS discharge, and the verification that both originated from the same cell by documenting a 10- to 20-ms pause in SS firing following the occurrence of a CS [38–40]. Following a preliminary separation and characterization of CSs and SSs, carried out online using Alpha Omega Engineering’s Multi Spike Detector (MSD) needed to direct the experimental approach, the definitive analysis was based on an offline approach as described in detail by Markanday and colleagues [41]. In short, a deep neural network was trained to use relevant features of PC recordings that allow fast and reliable identification of CSs as well as characterization of their morphology including the detection of their start and end times. The algorithm relies on dividing the record into a high-pass spectral band (300 Hz to 3 KHz) suitable for the characterization of action potentials recordings and a lower frequency band (30 Hz to 400 Hz) for the characterization of LFP signals.

### Data analysis

#### Saccade detection

All primary saccades of sizes between 13 to 17 degrees were detected with the help of a velocity threshold criterion of 30 deg/s. In order to detect corrective saccades with amplitudes varying from 0.2 to 2 deg, we used a more lenient velocity threshold of 10 deg/s in conjunction with the requirement of a duration longer than 10 ms.

#### Estimating the preferred direction and population response of complex spikes

Since saccades were only made horizontally left or rightwards, the directions were binned into 2 broad classes having a size of 180° each. Interestingly, we did not observe the expected CS discharge in response to the large retinal error caused by the initial target jump. However, in most PCs, and as also reflected by the population response (S2A Fig), we observed the strongest modulation in CS activity developing around the time of the saccade end. We therefore determined CS’s preferred primary saccade direction based on the probability of CS firing for each direction by calculating the total number of CSs fired during the *“early postsaccadic period”* of 0 to 100 ms after saccade end (brown shaded region in Fig 1C) and dividing them by the total number of saccades made in each direction. The direction with a higher mean probability of CS firing was defined as the preferred direction of the CS unit. For determining the preferred direction of corrective saccades, we calculated the firing rate during the *“postcorrective saccadic period”* of 0 to 100 ms from corrective saccade offset (purple shaded region in Fig 1C). To determine each PC CS’s preferred direction of performance errors, we used the *“precorrective saccadic period”* of −200 to 0 ms from corrective saccade onset, which was similar to the *“late postsaccadic period”* lasting from 50 to 250 ms from primary saccade offset (green shaded region in Fig 1C). In short, we refer to 3 postsaccadic periods of CS modulation: the primary saccade–related *“early postsaccadic period*,*”* the error-related *“late postsaccadic period” (or “precorrective saccadic period”)*, and the corrective saccade–related *“postcorrective saccadic period”*.

For computing the average discharge rate, we convolved each CS with a normalized Gaussian kernel of 5 ms standard deviation. CS population responses were estimated by calculating the mean of average firing rate of each PC.

#### Determining peak and trough times of complex spike firing rate

To capture the effects of saccade duration on the timing of CSs, we relied on the timing of the peak CS firing rate, as well as the timing of the trough. To calculate the peak time of the CS response, we randomly chose 50 PCs, each PC containing at least 10 trials for each duration bin (bin size = 5 ms) and calculated the mean firing rate and the time of the peak firing rate. By repeating this process 1,000 times, mean time of the peak and its 95% confidence intervals were estimated.

To estimate the time of the trough, which was well aligned to the end of saccades and marked the beginning of CS modulation (S2A Fig), we randomly selected 1,000 trials of each duration bin (bin size = 5 ms) across all cells and computed the mean discharge rate. Then, we fitted a second order polynomial to the baseline period (−200 ms to 0 ms from saccade onset), and a linear function from the time of the peak back to −45 ms to the trough (S2B Fig). The intersection of these 2 functions gave us an estimate of the trough time for each iteration. This process was repeated 1,000 times, and the mean timing of the trough and its 95% confidence interval were estimated.

#### Testing the encoding of different task parameters by complex spikes of individual Purkinje cells and their interdependence

To test for the encoding of a particular parameter/event (i.e., CP saccade offset relative to trial onset, primary saccade amplitude and direction, corrective saccade amplitude and direction, and error magnitude and direction) by CSs of individual PCs (see Fig 8B), the distribution of the values of a particular parameter (for example, CP saccade offset relative to trial onset) was obtained, and its median value was then used to divide the distribution into 2 groups. The mean CS firing rates were then calculated for the 2 groups for distinct periods of interest (period of interest for trial onset–related CSs: 150 to 250 ms from trial onset; for saccade amplitude and direction: 0 to 100 ms from primary saccade offset; for corrective saccade amplitude and direction: 0 to 100 ms from corrective saccade offset; for error magnitude and direction: 50 to 250 ms from saccade offset; see Fig 1C). The mean CS firing rates for the 2 groups for each parameter were then compared using a one-tailed Wilcoxon signed-rank test. For testing the directional preference for primary and corrective saccades, the mean firing rates from 0 to 100 ms from primary and corrective saccade offset, respectively, of the 2 opposite directions were compared using a one-tailed Wilcoxon signed-rank test. Differences were considered significant if *p* < 0.05.

To test the relationship between sets of parameters encoded by CSs of individual PCs, we conducted a cross-correlation analysis. For this, we assigned a value of “1” if a given PC significantly encoded (*p* < 0.05) a particular parameter and a value “0,” otherwise. This allowed us to obtain an m × n matrix in which m represents the PC ID and n represents the individual parameter. We then computed a cross-correlation between individual columns (i.e., between different parameters) of this matrix to obtain the matrix of cross-correlation coefficients shown in Fig 8C.

## Results

### Velocity-duration adjustments and endpoint variability during repetitive saccade task

In this study, we trained 2 rhesus monkeys to execute a long series of saccades toward a fixed target and back to the central starting location (Fig 1A). As expected, these stereotypic eye movements separated by short intertrial intervals of only 100 ms, caused a gradual decline in the vigor of saccades, arguably due to a gradual loss of interest over the course of trials. This “cognitive fatigue” [37], slowing down the pace of saccades, was compensated by an up-regulation of saccade duration sufficient to keep saccade endpoints within the required range of 2 deg around the target location at 15 deg eccentricity (see S1A and S1B Fig). Across all 160 recording sessions, we observed consistent effects as indicated by the averages (Fig 1B). Compared to the early (first 30) trials, the median peak velocity of both CF and CP saccades (Fig 1B, left panel) in the late (last 30) trials declined by 8.2% and 12.6% across all sessions, respectively (CF: early: 632.8 deg/s, late: 580.9 deg/s, Wilcoxon signed-rank test, *p* < 0.001, Z = 10.7; CP: early: 583.3 deg/s, late: 509.7 deg/s, Wilcoxon signed-rank test, *p* < 0.001, Z = 10.8). These changes were compensated by an increase of duration (Fig 1B, middle panel) of CF and CP saccades by 9.7% and 19.3%, respectively (CF: early: 39.9 ms, late: 43.8 ms, Wilcoxon signed-rank test, *p* < 0.001, Z = −10.7; CP: early: 43.3 ms, late: 51.7 ms, Wilcoxon signed-rank test, *p* < 0.001, Z = −10.8), thus keeping saccade amplitudes within an acceptable range of error (Fig 1B, right panel; CF: early: 14.7 deg, late: 14.6 deg, Wilcoxon signed-rank test, *p* = 0.02, Z = 2.2; CP: early: 14.8 deg, late: 14.9 deg, Wilcoxon signed-rank test, *p* = 0.03, Z = 2.1). Additionally, we also observed that the influence of fatigue, indicated by the amount of reduction in peak velocity, was stronger in the CP direction (12.6%) as compared to that of CF saccades (8.2%). Note that rewards followed the successful execution of CF saccades, whereas CP saccades were needed to get ready for a new trial, yet not followed by an immediate reward. Hence, the higher speed and shorter duration of CF saccades, their larger vigor, may be a consequence of more immediate reward expectations [42–46].

The natural endpoint variability in CF and CP saccades scattered around the target location (Fig 1B, right panel, S1C and S1D Fig) resulting in plenty of over- and undershoots that caused inward and outward retinal errors for each type of saccade. Therefore, for an exemplary session consisting of rightward CF and leftward CP saccades (see schematic diagram in Fig 1A), both inward and outward errors occurred in CF and CP direction. As will be discussed later, it is exactly this property that allowed us to tease apart the influence of primary saccades from their resulting errors on CS firing.

### Complex spikes carry information on the direction, amplitude, and duration of the primary saccade

Within a session, saccades could either over- or undershoot the target location resulting in both inward and outward retinal errors (Fig 1A). However, when comparing the CS firing rate for CF and CP saccades, we found clear saccade direction-dependent differences, regardless of the direction of the resulting errors. As demonstrated by the response of an example PC shown in Fig 1D, saccades to the right (CP in this case) were followed by a sharp increase in the CS firing during the postsaccadic period (0 to 100 ms after saccade end). Also, saccades in the opposite direction, CF saccades, were followed by an increased discharge. Yet, this increase was significantly weaker (peak firing rate ± SEM: CP = 5.97 ± 1.0 spikes/s; CF = 1.69 ± 0.5 spikes/s; Wilcoxon rank-sum test, *p* < 0.001, z = 4.20). We defined CSs’ preferred and antipreferred direction (PD_*ps*_ and PD_*ps*_+180°, respectively) for primary saccades (subscript: *ps*) based on their activity during the *“early postsaccadic period”* of 0 to 100 ms relative to saccade offset (see brown shaded region in Fig 1C). The overall CS firing across all 151 PCs in their PD_*ps*_ was clearly larger (Fig 1E, left) than to saccades made in the antipreferred direction (peak firing rate ± SEM: PD_*ps*_ = 1.76 ± 0.11 spikes/s; PD_*ps*_+180° = 1.07 ± 0.08 spikes/s; Wilcoxon signed-rank test, *p* < 0.001, z = 6.38). One may argue that trying to assess a preferred direction based on just 2 opposite horizontal directions may fail in many cases because of insufficient sensitivity. Yet, as demonstrated by the scatter plot of individual PCs’ CS mean firing rate in the antipreferred direction as a function of the CS mean firing rate in the preferred direction (Fig 1E, right), this was clearly not the case, with just a handful of CSs lying on the unity line. On the other hand, a plot of early postsaccadic CS firing for right versus left saccades (CF and CP combined; Fig 1F, left) did not yield consistent preferences (Fig 1F, left; peak firing rate ± SEM: left = 1.34 ± 0.10 spikes/s; right = 1.46 ± 0.10 spikes/s; Wilcoxon signed-rank test, *p* = 0.43, z = 0.78 and Fig 1F, right). The notion that this difference reflected saccade direction rather than a preference for CP saccades is indicated by the fact that other PCs could fire more CSs for CF saccades with no consistent preference for CP or CF saccades in our sample (peak firing rate ± SEM: CF = 1.33 ± 0.09 spikes/s; CP = 1.45 ± 0.10 spikes/s; Wilcoxon signed-rank test, *p* = 0.99, z = 9.38; S2D Fig). It could also be objected that the strong CS discharge observed in the *“early postsaccadic period”* might be a consequence of the large retinal error generated by the target jumps. However, in the vast majority of PCs (83%), we did not observe any significant modulation during the period between 50 ms from target jump time to the time of primary saccade whereas—as said before—we typically observed a clear response closely following the end of the saccade, suggesting that the observed CS discharge was related to the primary saccade itself. The possibility of whether this response may reflect a postsaccadic retinal error will be discussed later.

We further asked if the CS discharge for saccades made in the same direction yet starting from different positions differed from each other. To this end, we examined the CS responses of an exemplary PC, which could be tested for CF (and CP) saccades in both left and right directions during separate sessions. We found no difference in the CS peak firing rates for saccades with the same vectors but different points of origin (S2E and S2F Fig).

**Fig 2:**
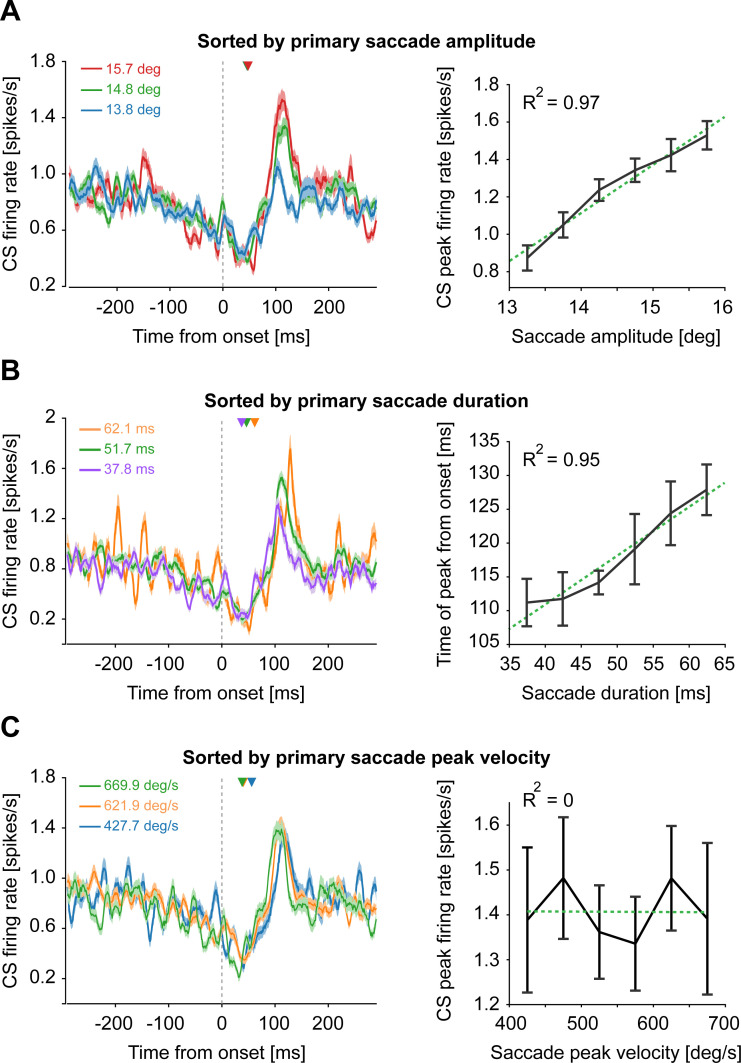
CSs encode primary saccade amplitude and duration but not peak velocity during the *“early postsaccadic period”*. **(A)** CS population response (mean ± SEM) aligned to the onset of all primary saccades (PD_*ps*_ and PD_*ps*_*+*180° combined) sorted by different amplitudes (left panel). The relationship between saccade amplitudes and peak firing rate of CSs is demonstrated with the help of a linear regression (right panel). **(B)** Population response (mean ± SEM) to saccades sorted by durations (left). The relationship between the timing of peak response (bootstrapped mean ± confidence intervals) and saccade duration is shown on the right. **(C)** Population response (mean ± SEM) to saccades with different velocities (left) and the corresponding regression plot (right). Green dotted lines represent the linear regression fits. The average saccade offset of each amplitude, duration, and peak velocity bin is denoted by inverted triangles in left panels. Note that the average onset of the upcoming corrective saccades occurred 349.2 ms after saccade onset. Underlying data available from the Dryad Digital Repository: [https://doi.org/10.5061/dryad.d51c5b03m]. CS, complex spike.

We next explored the effects of saccade amplitude on the firing probability of CSs. To this end, we exploited the natural variability of saccade endpoints within the fixation window of ±2 deg centered on the target location (for CF saccades) and the fixation point (for CP saccades). Because of the shortage of primary saccades with amplitudes larger than 16 deg and smaller than 13 deg, we confined the analysis to a range of saccades between 13 to 16 deg, divided into equally spaced bins (bin size: 0.5 deg) and pooled across PD_*ps*_ and PD_*ps*_+180° (i.e., CP and CF saccades in both left and right directions). As shown in Fig 2A, the maximum CS firing during the *“early postsaccadic period”* turned out to increase linearly with saccade amplitude (R-sq: 0.97; *p* = <0.001). To test the influence of saccade duration on CSs, we sorted the same pool of saccades into duration bins (range: 35 to 65 ms; bin size: 5 ms) and saw that longer duration saccades were associated with later population peak responses (Fig 2B; R-sq: 0.95; *p* = <0.001). Since the amplitude tended to increase with the duration of saccades, we also observed an increase in the CS firing for long-duration saccades. When aligning the CS population response to saccade offset (S2A Fig), we found that the postsaccadic increase in CS firing was preceded by a trough, a decrease in CS firing relative to the baseline level that at first glance seemed to indicate saccade end. This was indeed the case because also the timing of the trough (see Materials and methods for details; S2B Fig) proved to depend on saccade duration with trough times shifting with increasing saccade duration relative to saccade end (S2C Fig; R-sq: 0.86, *p* = 0.007). Surprisingly, considering the tight behavioral relationship between saccade velocity and duration, the firing probability of CSs turned out to be independent of saccadic peak velocity (Fig 2C; R-sq: 0; *p* = 0.99). We further investigated the effects of these kinematic parameters on CS firing individually in PD_ps_ and PD_ps_+180° and found similar results (S3 Fig). In sum, CSs in our sample carried information on the direction of the primary saccade, its amplitude, and duration, yet not saccade velocity.

### Complex spikes carry information on corrective eye movements

**Fig 3:**
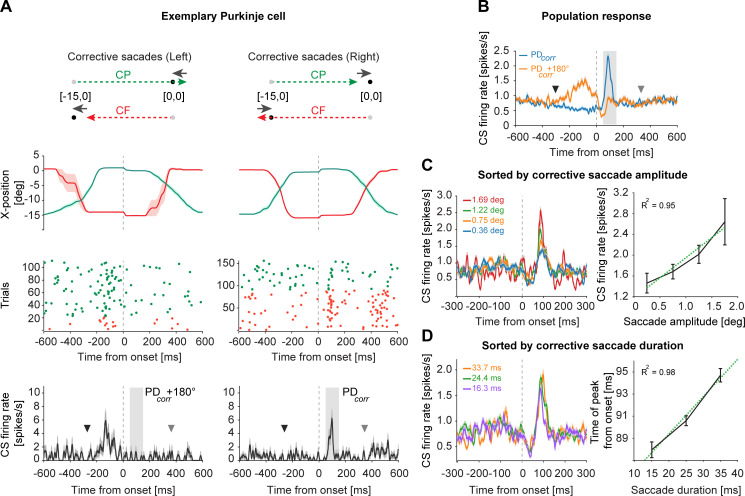
CSs encode corrective saccade direction, amplitude, and duration during the *“postcorrective saccadic period”*. **(A)** CS response of an exemplary PC to corrective saccades in leftward (left panels) and rightward direction (right panels). As illustrated by the schematic diagram on the top, supported by averaged (± SEM) saccade trajectories underneath, an experimental session with left CF (red) and right CP (green) saccades resulted in leftward corrective saccades that were made to correct the leftward error arising from CP saccades that “overshot” the fixation dot, and CF saccades that “undershot” the target location (left panel). The start and end positions of these corrective saccades were naturally different. Similarly, rightward corrective saccades resulted from overshooting CF and undershooting CP saccades (right panel). As seen in raster plots (middle panels) and the mean (± SEM) firing response of all trials (bottom panel), the peak firing rate of CSs was much larger for rightward (right panel) as compared to leftward (left panel) corrective saccades during the *“postcorrective saccadic period”* of 0–100 ms from corrective saccade end (gray shaded region). For each PC, CS’s preferred direction for corrective saccades (PD_*corr*_) was based on this period. Note that neither the different starting positions of corrective saccades nor the direction of preceding primary saccades influenced the CS firing. The average time of the preceding CF saccade offsets (= 272 ms) and the upcoming CP saccade onsets (= 362 ms), relative to corrective saccade onset, is marked by inverted black and gray triangles, respectively. **(B)** CS population response (mean ± SEM; *N =* 151 PCs) sorted by PC’s preferred (PD_*corr*_) and antipreferred (PD_*corr*_+180°) direction of corrective saccades based on *“postcorrective saccadic period”* (gray shaded region). Note that the CS activity during the *“precorrective saccadic period”* of approximately −200 to 0 ms from corrective saccade onset was used for determining the CS’s preferred direction of errors (PD_*error*_). The average time of the preceding CF saccades offsets (= 305 ms) and the upcoming CP saccade onsets (= 331 ms), relative to corrective saccade onset, is marked by inverted black and gray triangles, respectively. **(C)** CS population response (mean ± SEM, PD_*corr*_ and PD_*corr*_*+*180° combined) sorted by different amplitudes (left panel). The relationship between saccade amplitudes and peak firing rate of CSs is shown on the right. **(D)** CS response (mean ± SEM) to corrective saccades of different durations (left) and the corresponding relationship between the timing of peak response (bootstrapped mean ± confidence intervals) and saccade duration (right). Green dotted lines represent the linear regression fits. All data are aligned to corrective saccade onset. Underlying data available from the Dryad Digital Repository: [https://doi.org/10.5061/dryad.d51c5b03m]. CF, centrifugal; CP, centripetal; CS, complex spike; PC, Purkinje cell.

As a consequence of the inherent variability of saccade endpoints, causing retinal errors, saccades were followed by occasional secondary, corrective eye movements (amplitude: 0.2 to 2 deg) made toward the target location. As a matter of fact, we observed that the influence of these corrective saccades on CS firing was similar to the one of primary saccades. Fig 3A demonstrates the CS responses of an exemplary PC neuron (same PC as in Fig 1D) around the time of corrective saccades made during a single behavioral session. Trials, aligned with corrective saccade onset, were sorted according to the direction of the corrective saccade (left panels: corrective saccades to the left; right panels: to the right) regardless of their starting positions. No matter if the preceding primary saccade, ending approximately 272 ms before the corrective saccade onset, had been CP or CF, the CS firing rate increased within a period of about 100 ms from corrective saccade end (“*postcorrective saccadic period*”; see purple shaded region in Fig 1C). This is clearly depicted in the case of the exemplary PC’s CS response with a rightward preference for corrective saccades (Fig 3A) as exhibited by a strong discharge (peak firing rate: ± SEM: 6.25 ± 1.56 spikes/s) during the “*postcorrective saccadic period*” (Fig 3A; see raster plot and average CS response in the right panels), regardless of the preceding CF (red) or CP (green) saccades. In contrast, no clear modulation in CS firing was observed following corrective saccades to the left, the unit’s nonpreferred direction (peak firing rate ± SEM: 1.32 ± 0.92 spikes/s). Note, however, the increase in CS probability in the error period between primary saccade offset (inverted black triangle) and corrective saccade onset (Fig 3A, left panels); we will consider in more detail later.

As in the case of primary saccades, we compared the CS firing for corrective saccades made to the left and to the right, separated by using a bin size of 180°, in the “*postcorrective saccadic period”* of 0 to 100 ms from corrective saccade end. In other words, we only relied on the horizontal components of the corrective saccades in order to define their preferred and the antipreferred direction (PD_*corr*_ and PD_*corr*_+180°, respectively, subscript *corr*: *corrective saccades*). We did this for each CS unit. The preferred direction of the corrective saccades corresponded to the preferred direction for primary saccades in only 42% of the PCs, i.e., a percentage that is close to the one to be expected in case of a random relationship of preferred directions for primary and for corrective saccades. Fig 3B depicts the population responses for corrective saccades made in the preferred and the antipreferred directions. Similar to the CS population response for primary saccades made into their preferred direction, we observed a clear peak of CS firing in the PD_*corr*_ (peak firing rate ± SEM: PD_*corr*_ = 2.33 ± 0.14 spikes/s; PD_*corr*_+180° = 0.93 ± 0.07 spikes/s; Wilcoxon signed-rank test, *p* < 0.001, z = 7.74) during the *“postcorrective saccadic period”* (gray shaded region in Fig 3B). However, in contrast to the CS population response to primary saccades in PD_*ps*_+180° (Fig 1E, green trace), the CS population response to corrective saccades in the PD_*corr*_+180° direction exhibited a broader discharge during the *“precorrective saccadic period”* of 200 ms from corrective saccade onset (Fig 3A, bottom left panel, and Fig 3B, orange trace). We will discuss the basis of the CS modulation during this period in detail later after having first considered the amplitude tuning of the postcorrective saccade CS response.

To this end, we sorted all corrective saccades from all sessions in amplitude bins (bin size: 0.5 deg), pooling corrective saccades made in the PD_*corr*_ and PD_*corr*_+180°, and plotted the CS peak firing rate as a function of corrective saccade amplitude. As shown in Fig 3C, this resulted in a clear postcorrective saccade peak whose size depended linearly on the amplitude (R-sq: 0.95, *p* = 0.027). This was also true when looking at the CS population responses separately for the PD_*corr*_ and PD_*corr*_+180° (S4A–S4D Fig). Sorting corrective saccades based on duration, we observed that the peak of the CS population response occurred later if the corrective saccade took longer (Fig 3D; R-sq: 0.98, *p* = 0.081), a relationship that corresponds to the one for primary saccades. Further investigating the PD_*corr*_ and PD_*corr*_+180° individually for effects of saccade duration on the CS timing revealed similar patterns in both cases (S4E–S4J Fig). Moreover, even the timing of modulation onsets (“troughs”) shifted with corrective saccade durations (S4 and S4J Fig).

Our results on corrective saccades clearly indicate that CSs carry precise information on corrective saccade amplitudes and timing in the *“postcorrective saccadic period”* in a manner that is very similar to information on primary saccades in the “*early postsaccadic period*.”

### Complex spikes convey error-related information

As mentioned in the previous section, we also observed an increase in CS firing for corrective saccades in PD_*corr*_+180° −200 to 0 ms from corrective saccade onset (Fig 3B, orange trace).

**Fig 4:**
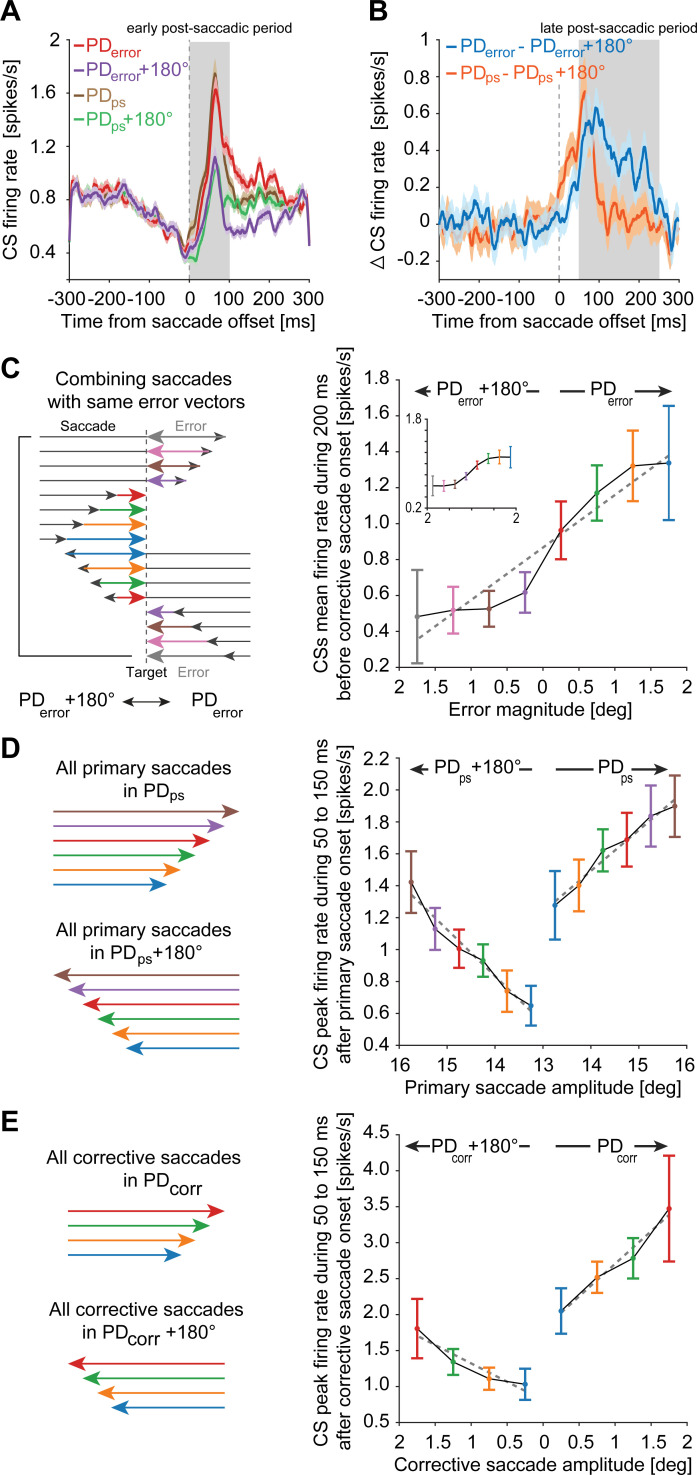
CSs encode the retinal error direction and magnitude during the *“late postsaccadic period”* in a manner different from primary and corrective saccades. **(A)** CS population response (mean ± SEM) aligned to the offset of primary saccades made in the preferred and antipreferred direction of errors (PD_*error*_, red; PD_*error*_+180°, purple) and primary saccades (PD_*ps*_, brown; PD_*ps*_+180°, green). Note the differences between CS responses in PD_*ps*_ and PD_*ps*_+180° are confined to the *“early postsaccadic period”* (gray shaded region). Note that the average onset of the upcoming corrective saccades was 305 ms relative to primary saccade offset. **(B)** Difference between the CS responses in PD_*error*_ and PD_*error*_+180° (blue) and PD_*ps*_ and PD_*ps*_+180° (orange). The influence of sorting CSs by PD_*error*_ can be seen as the separation of the curves during the *“late postsaccadic period”* of 50–250 ms from saccade offset (gray shaded region). **(C)** Left panel illustrates errors of different sizes (colored arrows) in PD_*error*_ and PD_*error*_+180° resulting from saccades of different amplitudes (black arrows) made in both directions. Saccades resulting in comparable error vectors were combined (for example, see gray arrows) to test the influence of size and direction of errors on CS firing. Right panel highlights the relationship between different error sizes and mean CS firing (± SEM) during the *“precorrective saccadic period*.*”* Note how the same error magnitude (see blue and gray) evokes a strong and a weak CS discharge depending on whether this error occurred in the preferred or antipreferred direction, respectively, thus showing a clear influence of error direction on CS firing. The inset demonstrates the same relationship when mean CS firing was calculated during the *“late postsaccadic period”* of 50–200 ms after saccade end. **(D)** Primary saccades of different amplitudes (colored arrows) in PD_*ps*_ and PD_*ps*_+180° (left panel) and their corresponding influence on the peak firing rate (mean ± SEM) during the *“early postsaccadic period”* (right). **(E)** Corrective saccades of different amplitudes (colored arrows) in PD_*corr*_ and PD_*corr*_+180° (left) and their corresponding influence on the peak firing rate (mean ± SEM) during the *“early postcorrective saccadic period”* (right). Dashed gray lines represent the linear regression fits. Underlying data available from the Dryad Digital Repository: [https://doi.org/10.5061/dryad.d51c5b03m]. CS, complex spike.

Could it be that this modulation that starts building up around the end of preceding primary saccades (Fig 3B, mean saccade end = 305.2 ms; inverted black triangle) is simply a delayed response to the preceding primary saccades as discussed in the earlier sections? Or was it related to retinal errors resulting from imprecise primary saccades prompting subsequent corrective saccades? To explore this alternative, we sorted all primary saccades recorded for a given PC according to the direction of the retinal error, i.e., the vector pointing from the primary saccade end toward the target location, ignoring primary saccade directions and their starting points (see illustration in Fig 1A). The error direction for which higher CS firing was observed in the *“precorrective saccadic period”* was labeled PD_*error*_ and the opposite direction, PD_*error*_+180°. For each PC, we calculated CS responses to primary saccades that caused errors in PD_*error*_ and PD_*error*_+180°, respectively, to compute CS population responses for these 2 directions. As shown in Fig 4A, the population averages differed in this *“early post saccadic period”* with the average in the preferred error direction characterized by a “burst-tonic” profile (red trace), starting to deviate from the population average for the opposite direction (purple trace) a few 10 ms after saccade offset, exhibiting a peak firing rate about 50 ms after saccade offset and staying elevated until well after 200 ms. Note that, also, the profile for the opposite direction showed an early peak while lacking the later tonic response component.

When sorted by the preferred direction of primary saccades, the population averages exhibited an early peak, significantly larger in the preferred saccade direction (Fig 4A, brown trace). Yet, the averages for the preferred and the antipreferred directions lacked any difference later, during the *“late postsaccadic period”* of 50 to 250 ms from primary saccade offset, falling back to baseline level already after only 113 ms. The contrast between the population averages sorted by retinal error as opposed to the averages sorted by primary saccade direction becomes particularly apparent when comparing the respective difference plots for the preferred and the antipreferred directions (Fig 4B), clearly demonstrating that the later modulation of CS firing is error specific and independent of the metric of the primary saccade. The possibility that this modulation might be influenced by the upcoming corrective saccades can also be excluded based on the fact that the average corrective saccade onset occurred much later, approximately 305 ms after primary saccade offset.

We wondered if the influence of error on the CS firing during this period is graded, reflecting not only an influence of direction but also of error magnitude. To find an answer, we sorted the CS population responses for corrective saccades into retinal error magnitude bins (bin size: 0.5 deg; Fig 4C, left) calculated for the *“precorrective saccadic period”* (200 ms before corrective saccade onset). As shown in Fig 4C, the mean firing rate of CSs increased with an increase in error magnitude in the PD_*error*_; however, in the opposite direction, CS firing decreased as the error magnitude increased (Fig 4C). In other words, a large error in PD_*error*_ triggered a stronger CS discharge, whereas the same magnitude of an error made in PD_*error*_+180° evoked a weaker CS discharge, thereby establishing a monotonic increase (R-sq: 0.92; *p* < 0.001) in CS firing as errors changed their magnitudes from antipreferred to preferred direction. We observed a similar pattern of CS firing rates during the *“late postsaccadic period”* of primary saccades, when sorted by error magnitude (see inset in Fig 4C; R-sq: 0.91; *p* < 0.001), clearly indicating that the *“precorrective saccadic period”* and *“late postsaccadic period’* shared the same error-related information and establishing that error influences CS firing in a graded manner.

As demonstrated earlier, primary saccade amplitude modulates CS firing in the “*early postsaccadic period*.*”* However, unlike the influence of error magnitude—which is opposite for errors in the preferred and the antipreferred direction—the influence on firing in the *“early postsaccadic period”* turned out to be the same for the 2 directions. For both primary saccades (Fig 4D) and for corrective saccades (Fig 4E), discharge increased with amplitude, no matter if saccades were made in the preferred or the antipreferred direction, respectively (PD_*ps*_: R-sq: 0.97, *p* < 0.001; PD_*ps*_+180°: R-sq: 0.96, *p* < 0.001; PD_*corr*_: R-sq: 0.97, *p* = 0.015; PD_*corr*_+180°: R-sq: 0.9, *p* = 0.053), with a relatively larger discharge in the preferred directions. However, note that the amplitude tuning could not be assessed across the whole range of amplitudes tested. This is clearly indicated by the fact that, in absolute terms, the CS firing associated with corrective saccades was clearly not weaker than the firing associated with the much larger primary saccades. Hence, in addition to direction and amplitude, the discharge in the “*early postsaccadic period”* is also determined by saccade type.

### Disentangling the influence of errors and saccade kinematics on postsaccadic complex spike activity

Our results clearly demonstrate that the early and the late postsaccadic periods of CS activity carry specific streams of information, related to saccade and error, respectively. However, given the close temporal proximity of these periods and the fact that we observed a peak CS discharge during the *“early postsaccadic period*,*”* regardless of whether the population averages were based on error or primary saccade direction (Fig 4A), we wondered if there may be an overlap of information encoding in these periods. Alternatively, could it be that the early postsaccadic CS response may simply reflect error rather than saccade direction and amplitude? To address these questions, we exploited the within-session endpoint variability in CF and CP saccades, due to under- and overshooting, resulting in oppositely directed retinal errors. We found that during the *“early postsaccadic period”* CSs preserved their specificity for the preferred saccade direction, even if the retinal error vectors pointed in opposite directions (see S5 Fig).

**Fig 5:**
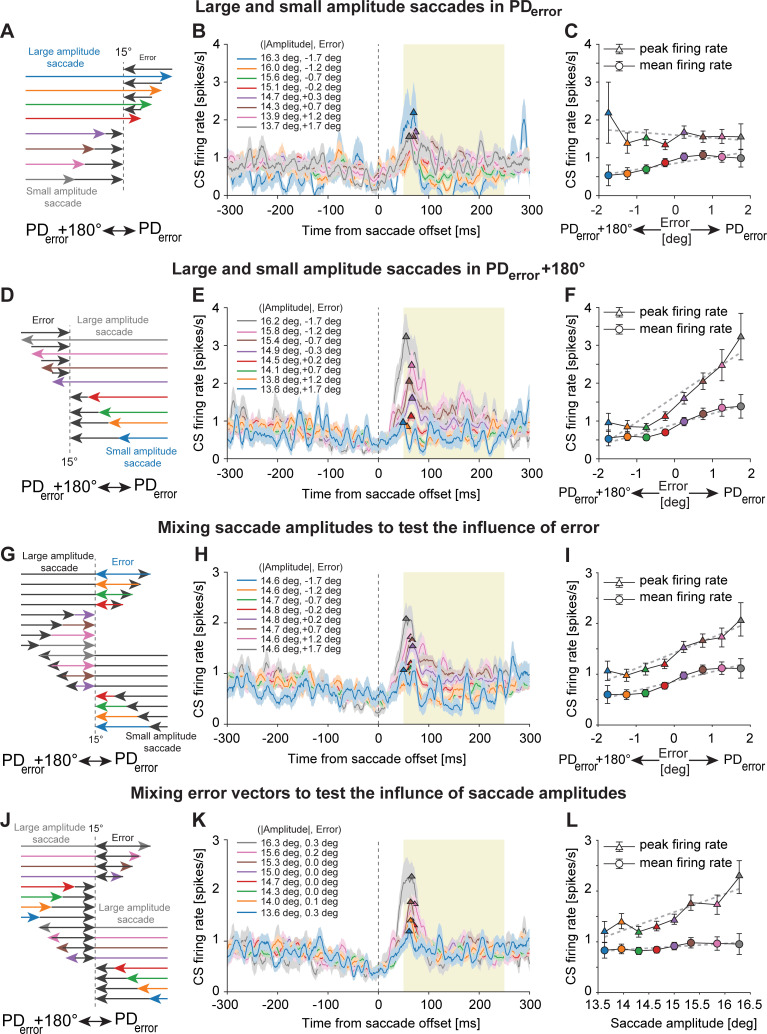
CS activity during the “*early postsaccadic period*” carries information on primary saccades and errors, whereas during the “*late postsaccadic period*,*”* it is mostly error driven. **(A)** Schematic diagram showing different amplitudes of primary saccades (colored arrows) made toward PD_*error*_ resulting from the sorting of errors (black arrows) occurring in PD_*error*_ and PD_*error*_+180° into bins of different magnitudes. Note how large and small amplitude saccades in PD_*error*_ were followed by errors in the opposite directions. Errors occurred in PD_*error*_+180° for large amplitude saccades, and for smaller amplitudes, they occurred in PD_*error*_. (B) CS population responses (mean ± SEM) to different amplitudes of primary saccades shown in A. Note how the peak firing of the population response (triangles) is observed much earlier toward the end of saccades. (C) Influence of different error sizes in PD_*error*_ and PD_*error*_+180° on peak CS firing (colored triangles, mean ± SEM) and mean CS firing (colored circles, mean ± SEM) calculated during the *“late postsaccadic period”* (yellow shaded region in B, E, H, and K). While the peak firing rate is meant to capture the saccade-related response, the mean firing rate tries to register error-related activity. (D) Schematic diagram showing different amplitudes of primary saccades (colored arrows) made toward PD_*error*_+180° and their respective errors (black arrows). (E, F) Same as B and C, respectively. (G) Schematic diagram showing primary saccades of different amplitudes (black arrows) pointing in PD_*error*_ and PD_*error*_+180° that resulted in different error sizes in both directions (colored arrows). Saccades with different amplitudes and pointing in opposite directions, albeit with the same error vectors (for example, see blue arrows), were combined to test the influence of errors on CS firing. (H) CS population response (mean ± SEM) to comparable amplitudes of primary saccades achieved by mixing saccades of different amplitudes as shown in G. (I) Same as in C. Note, however, the increase in CS peak firing rate despite comparable amplitudes of primary saccades indicating the influence of errors also in the *“early postsaccadic period*.*”* (J) Schematic diagram illustrating different amplitudes of primary saccades (colored arrows) in PD_*error*_ and PD_*error*_+180° and their corresponding errors (black arrows). To exclusively test the influence of saccade amplitudes, similarly sized saccades with errors in opposite directions (see gray arrows for instance) were combined, hence canceling the influence of errors. (K) CS population response (mean ± SEM) to primary saccades sorted by amplitudes, with mixed error directions. (L) Same as in C. Note how mixing the error directions led to the disappearance of differences between the CS population responses in the later error-related period. Also note that only the CS peak firing rates increased with saccade amplitudes but not the mean firing rate, suggesting the influence of saccade amplitudes in the *“early postsaccadic period*.*”* All data in B, E, H, and K are aligned to primary saccade offset (vertical dashed line). Dashed gray lines in C, F, I, and L represent fits based on linear regressions. Underlying data available from the Dryad Digital Repository: [https://doi.org/10.5061/dryad.d51c5b03m]. CS, complex spike.

We further investigated the CS population responses for saccades of varying amplitudes made in PD_*error*_ (Fig 5A–5C) and PD_*error*_ +180° (Fig 5D–5F) during the *“late postsaccadic period*.*”* To this end, for each PC, we sorted postsaccadic errors made in both PD_*error*_ and PD_*error*_ +180° into bins of different magnitudes (bin size = 0.5 deg), which resulted in the sorting of primary saccades of different amplitudes. However, given that the same error vector can result from primary saccades made in opposite directions (see over- and undershooting saccades followed by green arrows in Fig 1A), sorting errors by their magnitudes in each direction also resulted in the sorting of primary saccades such that their direction was either congruent or incongruent with PD_*error*_ and PD_*error*_ +180°. This allowed us to test the influence of amplitude of primary saccades made in PD_*error*_ and PD_*error*_ +180°, individually. Furthermore, depending on the direction and magnitude of errors, it also allowed us to test the influence of error on the primary saccade–related CS responses. As illustrated in Fig 5A, sorting errors in PD_*error*_+180° into bins of increasing magnitudes was accompanied by sorting of preceding primary saccades made in the opposite direction (i.e., PD_*error*_) in the order of increasing amplitudes (denoted by red, green, orange, and blue arrows) that overshot the target. On the other hand, sorting errors into bins of increasing magnitudes in PD_*error*_ resulted in small amplitude primary saccades (i.e., undershoots) in the same direction, but sorted by decreasing order of amplitudes (denoted by purple, brown, pink, and gray arrows). While the peak CS firing rate (denoted by triangles in Fig 5B and 5C) represents the influence of primary saccade amplitudes, the mean firing rate (denoted by circles in Fig 5C) represents the influence of retinal errors spread over this period. As demonstrated earlier in Figs 2A and 4D, one would also expect to see a linear increase in the CS peak firing rate as the saccade amplitude increases. However, unlike the analysis performed in Figs 2A and 4D in which the direction of resulting retinal errors was ignored, we found that the influence of saccade amplitudes (see peak firing rates in Fig 5B and 5C) on CS firing was strongly affected when the direction of subsequent errors was considered. For instance, a large error in PD_*error*_, resulting from a small amplitude saccade (Fig 5A, see gray arrow) made in the same direction, slightly increased the peak CS firing for this saccade amplitude (Fig 5B and 5C). Given the linear relationship of saccade amplitudes to CS firing (Fig 4D), one would have expected to see a decrease in peak firing rate as the saccade amplitude decreases. However, as indicated by a poor slope of regression in Fig 5C (R-sq = 0.13; slope = −0.08), this was not the case. On the other hand, the influence of different error sizes can still be seen as a monotonic increase in the mean CS firing rate as error vectors shift from PD_*error*_+180° toward PD_*error*_ (R-sq = 0.82; slope = 0.16; Fig 5C). For large and small amplitude saccades made in PD_*error*_+180°, error directions were reversed (Fig 5D). Consequently, large amplitude saccades in PD_*error*_+180° (for instance, gray arrow in Fig 5D) followed by errors in PD_*error*_ exhibited an elevated peak firing rate (Fig 5E), an effect that may have been facilitated by errors occurring in their preferred direction. Similarly, CS response to small amplitude saccades may have been further suppressed by the influence of subsequent errors made in the PD_*error*_+180°. This interpretation is well captured by positive regressions for both peak firing rates (R-sq = 0.88; slope = 0.67) and mean firing rates (R-sq = 0.92; slope = 0.28) in Fig 5F. To test the influence of errors on the peak and mean CS discharge during the *“late postsaccadic period*,*”* not confounded by any influence of saccade metrics, we combined saccades resulting in comparable error vectors. To this end, we pooled saccades independent of saccade direction (left or right, CF or CP) and amplitude, but with comparable error vectors (Fig 5G, see saccades with error vectors labeled as blue arrows for instance). Error vectors were considered comparable if they fell in 1 out of 8 error vector bins (error direction is indicated by the sign of each value. Positive for PD_*error*_ and negative for PD_*error*_+180°). As shown in Fig 5H, the resulting error vector–specific CS profiles for the 8 bins diverged in the *“late postsaccadic period*.*”* Both peak and mean firing rates modulated with error vector size (Fig 5I, peak firing rate: R-sq = 0.92, slope = 0.31; mean firing rate: R-sq = 0.91, slope = 0.19). As the mean saccade amplitudes for the 8 error vector bins were statistically indistinguishable (Kruskal–Wallis test, χ^2^ = 11.15, df = 7; *p* > 0.05), the divergence of curves must be due to differences in error size, suggesting that CS responses in the *“late postsaccadic period”* are primarily—if not exclusively—error driven. While this holds for the larger part of this period, trying to annihilate the impact of the error on the CS responses prompts a qualification of this conclusion. When pooling saccades that result in errors in opposite directions (Fig 5J; for example, large-amplitude saccades represented by gray arrows, with opposite error vectors, were combined), in order to annihilate the potential influence of errors during the “*late postsaccadic period*,” an influence of saccade amplitude on the peak firing rates (R-sq = 0.8, slope = 0.36) early in this period became apparent. On the other hand, the later, much longer part of the CS discharge in the “*late postsaccadic period*,*”* largely determining the mean CS firing rate, remained unaffected by saccade amplitude (R-sq = 0.73, slope = 0.06). Taken together, firing in the early part of the “*late postsaccadic period”* exhibits a mixed influence of saccade amplitude and retinal error, whereas the later, much longer part of the response is exclusively error driven. A clear impact of saccade amplitude and direction during the *“early postsaccadic period”* is also demonstrated by distinguishing large and small amplitude saccades (with on average comparable error vectors) in PD_*ps*_ and PD_*ps*_*+*180°, separately (S6 Fig).

Finally, we performed a multiple regression analysis on the influence of saccade amplitude and error on the CS discharge in 2 narrow nonoverlapping periods of 40 to 80 ms and 100 to 250 ms from saccade offset, chosen such as to separate the early and the late postsaccadic periods distinguished in the preceding paragraph. Fully consistent with the conclusion based on the analysis summarized in Fig 5, the multiple regression analysis documented that the CS activity during the *“early postsaccadic period”* is modulated by changes in saccade amplitude and error (S7A Fig), whereas the CS response during the “*late postsaccadic period”* is mostly error driven (S7B Fig).

### Complex spike duration encodes error- and saccade-related information

**Fig 6:**
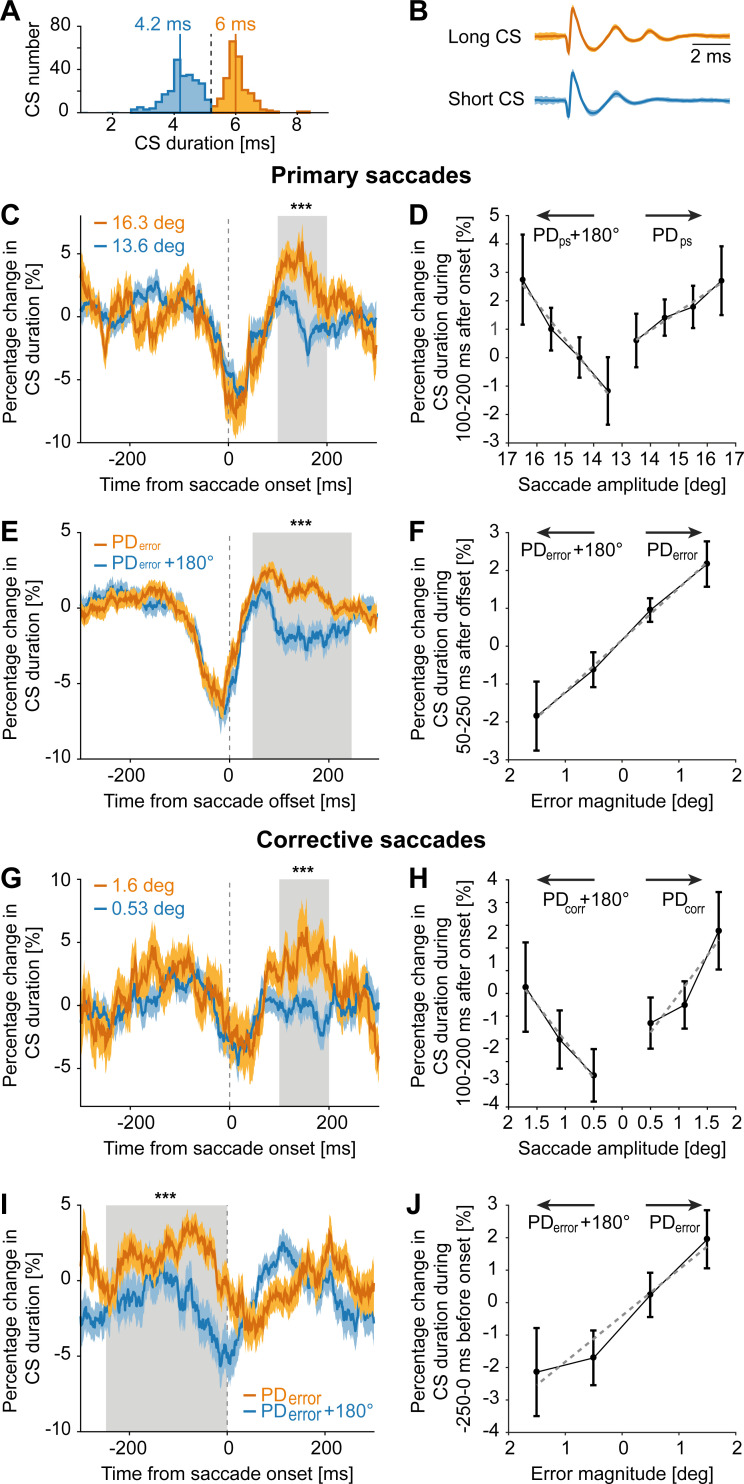
Changes in CS duration encode changes in primary and corrective saccades as well as errors. **(A)** Histogram showing a bimodal distribution of CS durations in an exemplary PC. For illustration purposes, we separated the distribution into long and short duration CSs by eye (vertical dashed line). Solid vertical lines represent the median value of short (blue) and long (dark yellow) duration CSs. **(B)** Averaged (± SEM) waveforms of long and short duration CSs. **(C)** Population response (mean ± SEM) showing percentage change in CS duration aligned to primary saccade onset, for large (dark yellow) and small (blue) amplitude saccades. The population response was obtained from running averages of percentage change in CS duration, computed for every 50 ms bins, relative to the mean CS duration of each PC. **(D)** Percentage change in CS duration during the 100–200 ms period from primary saccade onset (gray shaded region in C) relative to saccade amplitudes in PD_*ps*_ and PD_*ps*_+180°. **(E)** Percentage change in CS duration for primary saccades made in PD_*error*_ and PD_*error*_+180°. Data aligned to primary saccade end. **(F)** Percentage change in CS duration relative to error magnitudes in PD_*error*_ and PD_*error*_+180° during the 50–250 ms period from saccade offset. **(G, H)** Same as C and D, except for corrective saccade. **(I)** Percentage change in CS duration for corrective saccades in PD_*error*_ and PD_*error*_+180°. **(J)** Percentage change in CS duration relative to error magnitudes in PD_*error*_ and PD_*error*_+180° during the −250–0 ms period from corrective saccade onset. Data in G and I aligned to corrective saccade onset. Error bars and dashed gray lines in D, F, H, and J represent the SEM and fits based on linear regression, respectively. Significant differences between CS responses in **C**, E, G, and I are indicated by asterisks. Underlying data available from the Dryad Digital Repository: [https://doi.org/10.5061/dryad.d51c5b03m]. CS, complex spike; PC, Purkinje cell.

It has been argued earlier that not only the probability of CSs firing but also systematic changes in CS duration, the latter being dependent on the “state” of the olivary neurons determining the strength of the climbing fiber input [47,48], carry behaviorally relevant information necessary to drive motor learning [26,27]. To test the relevance of CS duration in our task, we measured the duration of individual CSs fired by each PC as the time between CS start and end, by deploying an interactive deep neural network [41]. As shown for an exemplary PC neuron (Fig 6A), we found a bimodal distribution of CS durations for a single behavioral session. Taking a closer look at the CSs in the 2 modes revealed that the longer duration CSs (mean duration of CS: 6 ms) were characterized by a waveform with an additional spikelet at the end, not exhibited by CSs in the short duration mode (mean duration of CS: 4.2 ms). There was no change in the shape of the initial fast-spiking component (Fig 6B).

To investigate task-related changes in CS duration, we computed a running average (bin size: 50 ms) of percentage change in CS duration relative to mean CS duration of an individual PC and estimated a population-based percentage change in CS duration. This population measure showed a conspicuous drop relative to baseline (peak change from baseline: 7.5%) in the perisaccadic period. In the subsequent phase, the population CS duration increased again, reaching a maximum value of 1.6%, about 100 ms after primary saccade end. When comparing the trajectory of duration changes with the one for CS firing rate (S8 Fig), it became clear that the two were obviously yoked, with decreases in duration roughly paralleling decreases in CS probability and vice versa. Therefore, given that the postsaccadic CS discharge is modulated by changes in saccade amplitude and error size, we asked if CS duration carried this information in similar periods. To this end, we compared mean CS duration changes in the early and late postsaccadic periods (gray shaded regions in Fig 6) for changes in primary and corrective saccade amplitudes, as well as errors.

When sorting primary saccades by their amplitudes, it became apparent that the CS duration changes for larger amplitude saccades (mean amplitude: 16.4 deg) differed from those for smaller amplitude saccades (mean amplitude: 13.7 deg) in the postsaccadic period (Wilcoxon signed-rank test, *p* < 0.001, z = 8.72), of 100 to 200 ms after saccade onset, i.e., approximately 50 to 100 ms after saccade end (Fig 6C). As shown in Fig 6D, the percentage change in CS duration during the postsaccadic period increased with primary saccade amplitude (bin size: 1 deg), independent of saccade direction (PD_*ps*_: R-sq = 0.98, *p* = 0.011; PD_*ps*_+180°: R-sq = 0.99, *p* = 0.007), a pattern very similar to the amplitude tuning obtained for CS firing rates (Fig 4D). Distinguishing primary saccades based on their preferred error direction (i.e., PD_*error*_ versus PD_*error*_+180°; Wilcoxon signed-rank test, *p* < 0.001, z = 12.29; Fig 6E), we observed that CS duration grew in the error period for saccades causing larger errors in the preferred error direction and dropped in the opposite direction (Fig 6F; R-sq = 0.99, *p* = 0.0014), a pattern reminiscent of the one observed for CS firing rates in response to error magnitudes (Fig 4C). Similar to the dependence of CS firing on the size of corrective saccades (Fig 4E), we found that CS durations increased with corrective saccade amplitude (Fig 6G, Wilcoxon signed-rank test, *p* < 0.001, z = 5.1), regardless of their direction (i.e., PD_*corr*_ and PD_*corr*_+180°; PD_*corr*_: R-sq = 0.89, *p* = 0.215; PD_*corr*_+180°: R-sq = 0.99, *p* = 0.069; Fig 6H). Note that for the comparison of mean CS duration changes for different amplitudes of primary and corrective saccades (gray shaded regions in Fig 6C and 6G), we slightly delayed the beginning of these periods, relative to the *“early postsaccadic period*,*”* in order to highlight subtle changes in CS duration. Finally, the influence of error magnitude on CS duration before the corrective saccade (Fig 6I; Wilcoxon signed-rank test, *p* < 0.001, z = 7.57) was similar to the one for primary saccades in preferred and antipreferred error directions (Fig 6J; R-sq = 0.95, *p* = 0.026). Together, these findings clearly suggest that CSs convey saccade- and error-related information by duration changes that parallel changes in CS firing rates.

### Trial onset evoked responses of complex spikes

**Fig 7:**
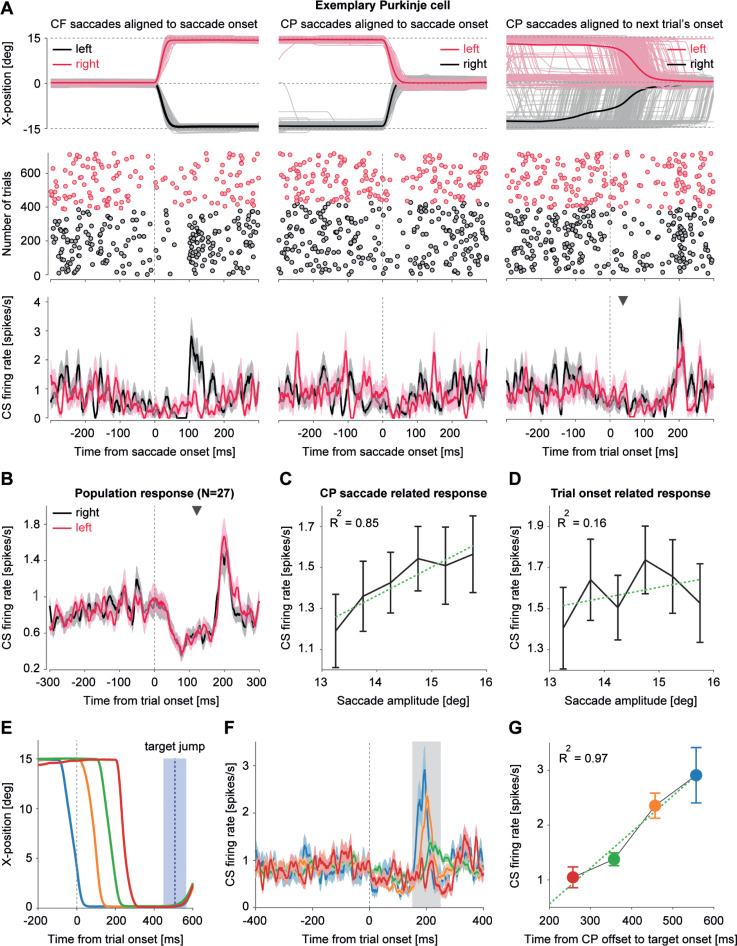
A trial onset–related CS discharge seems to predict the upcoming events. **(A)** CS response of an exemplary PC neuron tested in both, left and right, CF (left column) and CP (middle column) directions. Data are aligned to saccade onset. CP saccades and the CS responses aligned to the onset of the next trial are shown in the rightmost column. Despite the variability of saccades, note how precisely CS are accumulated around 200 ms from trial onset. Upper row: Individual saccades are shown as thin lines. Thick lines represent average saccade trajectories. Middle row: raster plot of CSs. Lower row: mean (± SEM) CS response for each condition. The average onset of all CP saccades (= 111 ms) relative to trial onset is marked by the inverted black triangle. Note that the average onset of the upcoming CF saccades occurred after 669 ms from trial onset. **(B)** Trial onset evoked CS response (mean ± SEM) of all PCs (*N =* 27) tested for saccades in both directions. Red and black traces correspond to the left and right position of the trial onset related fixation dot relative to the eye position at the end of the previous CF saccade, respectively. The inverted black triangle denotes the average CP saccade onset relative to the trial onset. **(C)** Change in peak firing rate of CS population response (*N* = 151, mean ± SEM), aligned to CP saccade onset, relative to changes in CP saccade amplitudes (mean ± SEM). **(D)** Change in peak firing rate of CS population response (*N* = 151, mean ± SEM), aligned to next trial’s onset, relative to changes in CP saccade amplitude (mean ± SEM). Note how the same CSs lose information on saccade amplitudes when aligned to trial onset. **(E–G)** CP saccades (average trajectories) sorted by their time of arrival at the fixation dot, relative to the time of target jump and corresponding changes in peak firing rate of the CS population response (mean ± SEM). Mean ± SD of target jump times is represented by the blue vertical dotted line and shaded region in blue, respectively. Error bars represent ± SEM around the mean. Dotted green line represents the linear regression fit. Underlying data available from the Dryad Digital Repository: [https://doi.org/10.5061/dryad.d51c5b03m]. CF, centrifugal; CP, centripetal; CS, complex spike; PC, Purkinje cell.

When analyzing CSs of individual PCs, we found that in a subset of PCs (*N =* 73 out of 151), the probability of CS firing increased more strongly before an upcoming saccade when aligned to the appearance of the central fixation dot as compared to aligning trials to the onsets of the preceding CP saccades. The appearance of the central fixation dot, marking the beginning of the new trial, also served as a target for CP saccades. However, due to the repetitive nature of the task accompanied by very short intertrial intervals, some CP saccades were also anticipatory such that they arrived at the location of the fixation dot before its appearance. Fig 7A shows an example of one of those few PCs (*N* = 27) that could be held long enough to record CSs in both left and right CF directions, in separate sessions. This cell fired CSs following left but not right CF saccades (Fig 7A, left column). In the case of CP saccades (Fig 7A, middle column), we observed only a very weak modulation during the postsaccadic period. However, the CS firing rate was clearly enhanced when aligning trials with CP saccades to the onset of the upcoming CF trials (marked by the appearance of the central fixation dot) with a relatively sharp peak in CS firing at 200 ms after trial onset, regardless of direction (Fig 7A, right column; peak firing rate ± SEM: left = 3.40 ± 0.39 spikes/s; right = 2.40 ± 0.46 spikes/s; Wilcoxon signed-rank test *p* = 0.40, z = 0.83). Considering the large temporal scatter of the preceding CP saccade endpoints relative to the onset of the upcoming CF trial onset, the temporal precision of this CS discharge, resulting in a sharp peak, clearly argues against the possibility that it might be a late reflection of the preceding CP saccades. Could it be a response to the large visual error due to the appearance of the central fixation dot relative to the position of the eyes at the end of CF saccades? This possibility can be safely overruled because of the lack of directional specificity of the CS peak discharge (Fig 7A, right column), assuming that any sensitivity to retinal error should be directional. We pooled CS responses of all 27 PCs (Fig 7B), from which data on both left and right CF (and CP) saccades were available and found no difference between the size of the CS peak discharge rates in the trial onset aligned CS population responses for the 2 directions (peak firing rate ± SEM: left = 1.51 ± 0.18 spikes/s; right = 1.67 ± 0.20 spikes/s; Wilcoxon signed-rank test, *p* = 0.37, z = 0.89). If these peaks were related to the CP saccades preceding the upcoming trial, one might expect to see an influence of CP saccades on all task-related CSs fired at about the time at which we observed the CS peaks tentatively related to trial onset. Moreover, this influence would be expected to be CP saccade amplitude related. This was clearly not the case as documented by a consideration of pooled CS responses of all 151 task-related PCs. Although their CSs firing rate clearly modulated with CP saccade amplitudes (Fig 7C; R-sq = 0.85, *p* < 0.01) when aligned to CP saccade offset during the *“early postsaccadic period*,*”* the same CSs when aligned to trial onset exhibited no clear relationship to CP saccade amplitudes made toward the central fixation dot (Fig 7D; R-sq = 0.16, *p* = 0.44). Finally, we sorted the CP saccades according to their arrival times relative to the time of the onset of the upcoming trial (Fig 7E). As shown in Fig 7F and 7G, the “onset” peaks were absent in the case of late CP saccades that arrived only shortly before the next jump of the target to an eccentric position. On the other hand, the peaks were maximal in the case of early CP saccades with a gradual change between these 2 extremes (Fig 7G; R-sq = 0.97; *p* = 0.016). In sum, these results indicate that the increase in CS firing around 200 ms after trial onset is related neither to the retinal error nor to the kinematics of the preceding CP saccade. Rather, it may reflect a prediction of the upcoming event, i.e., the time of the target jump and/or preparation of the behavior required.

## Discussion

**Fig 8:**
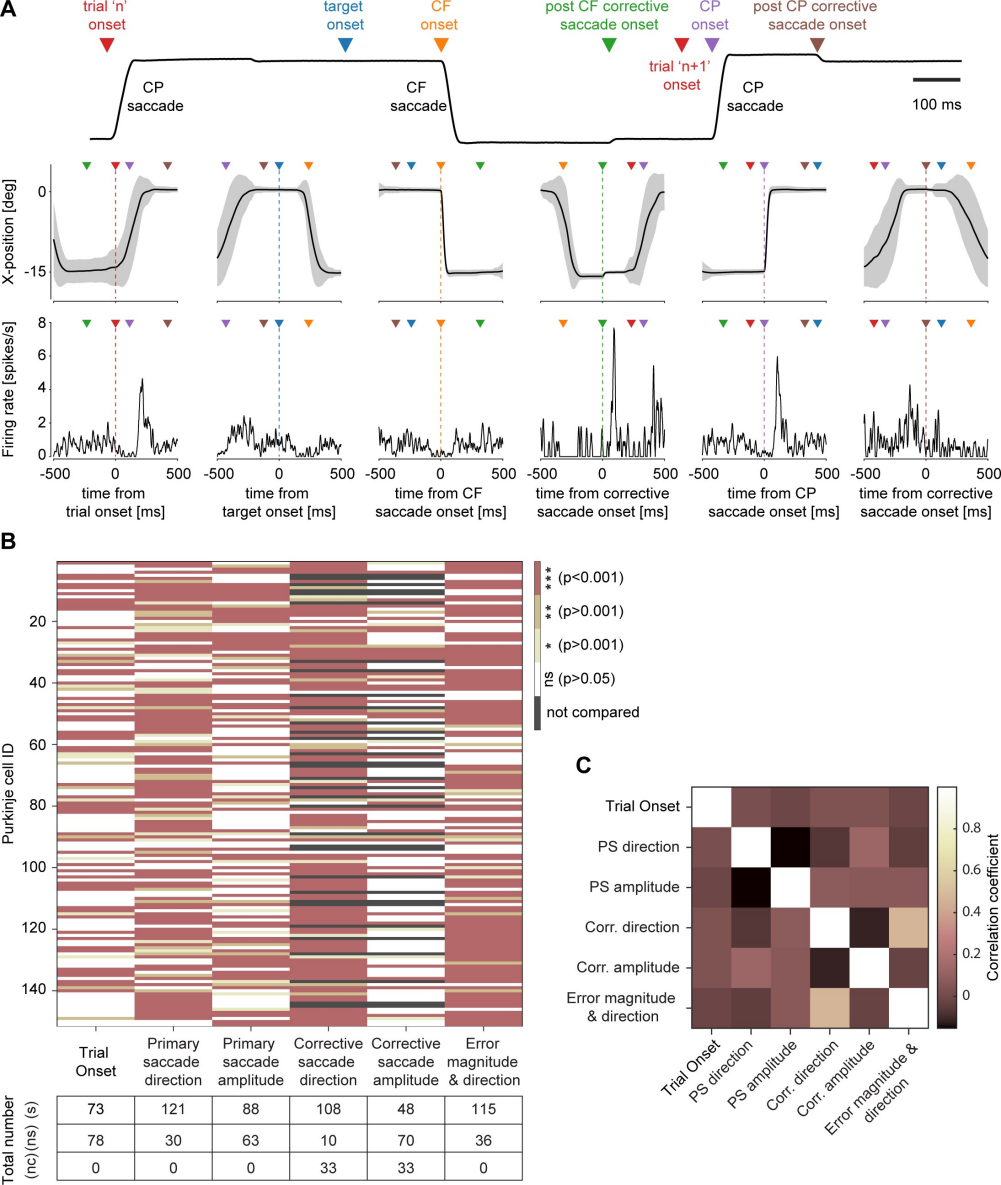
Encoding of different task parameters by individual PCs. (**A)** The upper panel illustrates different events (indicated by inverted triangles of different colors) in their temporal order within a trial, relative to the saccade behavior (solid black trace). Middle and bottom panels show the averaged horizontal eye position traces and the averaged CS response of an exemplary PC aligned to each of the events (colored vertical dashed lines) shown in the upper panel. The relative timing of the preceding and upcoming events is also shown as inverted colored triangles. **(B)** The number of rows in the figure corresponds to the ID of the PC tested, and each column corresponds to the type of parameter/event encoded. Different colors represent the range of *p*-values obtained from comparisons of different values of each parameter (see Materials and methods for more details). Cells that fired less than 10 CSs during the period of interest were not compared (shown as dark gray colored boxes). A summary of the total number of PCs encoding each type of information is provided in the table below. **(C)** A cross-correlation matrix summarizing the relationship between the encoding of different parameters encoded by PCs. The strength of the relationship between each comparison is determined by the value of correlation coefficients, which is represented by the color of each pixel. Note that we found only a moderate correlation (cross-correlation coefficient = 0.46) between corrective saccade direction and error magnitude and direction. In all other cases, we did not observe any meaningful relationships between parameters encoded by the individual PCs (cross-correlation coefficient < 0.2). Underlying data available from the Dryad Digital Repository: [https://doi.org/10.5061/dryad.d51c5b03m]. CF, centrifugal; CS, complex spike; nc, not compared; ns, nonsignificant; PC, Purkinje cell; PS, primary saccade; s, significant.

The purpose of this study was to investigate if CSs are capable of carrying different streams of behaviorally relevant information in a multiplexed manner. By scrutinizing whether CS responses evoked in a demanding saccade paradigm causing cognitive fatigue are related to different events (see Fig 8A for an exemplary PC), we were able to demonstrate that many seemingly “spontaneously firing” CSs actually transport information on several behaviorally relevant parameters.

### Complex spikes encode saccade-related parameters

When aligning the CS discharge to primary saccades, we observed a strong and temporally precise accumulation of CSs within a 100-ms period starting precisely at the end of saccades. This strong modulation was clearest for saccades made in 1 of 2 horizontal directions, specific to the respective PC. Since we tested CF and CP saccades in only left and right horizontal directions, it is important to note that our consideration of the directionality of the saccade-related CS responses must be very coarse. Therefore, in some cases (30 PCs; see Fig 8B) in which we did not find clear differences between CS responses in the 2 saccade directions, we probably missed the preferred direction because it may have deviated too much from the horizontal. In any case, CS firing increased linearly with the amplitude of the saccade made in our admittedly coarse estimate of the preferred direction. The subtle changes in saccade duration due to fatigue were paralleled by shifts in the latency of the peak of the modulation and by shifts in the onset of this modulation. At first glance, one may feel tempted to argue that CSs occurring during this *“early postsaccadic period”* may only convey error-related information and that the observed increase in the CS firing in response to saccade amplitudes may simply reflect retinal errors emerging from imprecise saccades possibly growing with saccade amplitude. However, there are several reasons to discard this interpretation. First, we found a strong relationship between the end of the primary saccade and the onset of CS modulation with increases in saccade duration associated with later modulation onset (Fig 2B, S2A–S2C Fig). Second, in population averages in which the 2 error directions were balanced, we still found clear saccade-related responses in this *“early postsaccadic period”* (Fig 5J–5L, S6 Fig). Moreover, these responses were saccade amplitude dependent. On the other hand, changes during the “*late postsaccadic period”* only appeared when CSs were sorted by error direction (Fig 5G–5I). Third, also corrective saccades (<2 deg) exhibited an increased CS firing within a 100-ms period from saccade offset that showed a saccade amplitude (Fig 3C) and duration (Fig 3D) dependence, fully analogous to the one for primary saccades. Note that the estimated preferred directions of primary and corrective saccades matched in only 42% of the PCs. However, this weak relationship is not surprising as it may simply reflect the consequences of using a large direction bin that ignores the vertical components of saccades, which can largely differ in the 2 types of saccades. For example, given that the target appears at 15 deg to the right, if we assume that the PD_ps_ of a given CS unit is to the right, then a primary saccade with a tiny overshoot of 1° with a tiny vertical component of 1° will probably maximally drive the unit. However, the resulting error vector and, consequently, the corrective saccade will have a direction of 45°, hence substantially deviating from the assumed preferred direction of the CS unit. Assuming that corrective saccades will finally lead to target fovealization, one would not expect any discharge due to a remaining directional retinal error. Hence, we are confident that the CS response during the “*early postsaccadic period”* reflects the metric of saccades. This notion is in accordance with previous work on reaching arm movements of monkeys demonstrating an influence of movement direction and kinematics on CSs [10,49] and with the work on saccadic adaptation [18] that demonstrated the build-up of CS modulation with changes in saccade amplitude paralleling the disappearance of error. However, given that the influence of the primary saccade on the CS discharge appears at the end of the saccade, it cannot be causally related to the programming of this saccade. On the other hand, it is early enough to influence the impact of information on the retinal error, dominating later parts of the CS response. We suggest that this saccade-related response is in fact an efference copy of the motor command whose purpose will be discussed in the last section.

### Complex spikes encode error-related information

The fact that we found clear nonvisual saccade-related responses does not refute the idea that CSs encode retinal errors. We could clearly identify error direction–dependent changes in CS firing at the level of individual PCs. However, they occur later than the ones related to saccade kinematics with some overlap notwithstanding. Since we used the horizontal component of these errors to divide them into 2 large bins (bin size: 180 deg), the possibility that the CS’s true preferred direction for some errors may have been missed cannot be completely ruled out. In fact, the mixed responses to saccade and error parameters, which we observe during the *“early postsaccadic period”* (Fig 5, S7 Fig), might be a consequence of the potential influence of a weak and temporally imprecise CS response to retinal errors spread out during the postsaccadic period. These overlapping responses notwithstanding, the lack of a difference in the CS response to mixed errors during the *“late postsaccadic period”* (Fig 5J–5L, S6A Fig), in conjunction with the result of our multiple regression analysis (S7 Fig), clearly speaks in favor of 2 separate periods for encoding saccade- and error-related information. In CS population averages, the error-related influence started around 50 ms after saccade offset and lasted for more than 200 ms, i.e., at corrective saccade onset. In fact, this is the same period in which Herzfeld and colleagues [7,8] reported error-related CS activity. We observed a linear increase in the CS firing rate with error magnitude within a small range of magnitudes (<2 deg) in the preferred direction, while previous studies reported an absence of error magnitude tuning [14,50]. However, this is not necessarily inconsistent as we did not consider larger errors for which the tuning we describe might exhibit saturation.

### Encoding of saccade- and error-related information by complex spike duration

An additional role of resurgent sodium currents at the postsynaptic PC influencing the number of spikelets in the CS notwithstanding [51,52], the duration and morphology of CSs are mainly determined by the duration and temporal structure of action potential bursts fired by the presynaptic olivary climbing fibers [48]. The latter reflects the state of the inferior olive as set by gap junctions controlling the communication between neighboring cells. Longer duration axonal bursts are thought to translate into CSs with more spikelets and overall longer duration [25,29,39,48]. Increasing the duration of CS will lead to more influx of calcium, boosting long-term depression at parallel fiber-PC synapses, believed to be the major basis of cerebellar learning [26,27]. In accordance with this framework, Yang and Lisberger [26] could demonstrate that smooth-pursuit adaptation, a variant of oculomotor learning, was accompanied by changes in CS duration. In contrast, previous attempts to identify corresponding correlations between CS duration and saccadic adaptation failed [8,20]. However, in accordance with previous findings on smooth-pursuit eye movements [27], we could establish clear saccade-related changes in CS duration accompanying changes in CS firing rates (Fig 6). Yoked changes in CS rate and duration reflected both information on saccade metrics and retinal errors. CS duration changes were typically based on the addition or omission of terminal spikelets. These findings support the notion that the control of PC calcium levels, underpinning synaptic plasticity, deploys concerted changes in CS rate and duration.

Changes in saccade-related CS firing were not only confined to increases in rates, accompanied by changes in CS duration, but also involved a conspicuous decreased component around the time of the saccade (S8 Fig). The phenomenology of this pause in CS firing, accompanied by a drop in CS duration, is particularly intriguing as its time course is strikingly similar to the time course of saccadic suppression of perception, both spanning a perisaccadic period of approximately −75 to +85 ms relative to saccade onset. Saccadic suppression is thought to have a neural correlate in the superior colliculus, where neurons in the intermediate superior colliculus exhibit a suppression of their discharge that parallels the perceptual phenomenon [53]. Given that climbing fibers in the OMV receive their visual input probably primarily from the superior colliculus [54–59], we speculate that the perisaccadic suppression of CS activity might be handed over from the superior colliculus for whatever purpose.

### Complex spikes predict upcoming events

We found that a large proportion of task-related PCs showed a sharp increase in CS firing around 200 ms after trial onset (see table in Fig 8B), i.e., at a time the monkey got ready for a new target-directed CF saccade. Our analysis of a subset of PCs, for which data on CF and CP saccades in both left and right directions was available (see S1 Table), revealed that this modulation in CS activity was insensitive to the direction or amplitude of preceding CP saccades. Aligning these responses to different events other than the onset of the new trial only compromised the strength of the CS response (Fig 7A), supporting the link to trial onset, marked by the reappearance of the central fixation dot. The reappearance of the fixation dot was followed by target jumps 400 to 600 ms later, to which the monkeys responded with target-directed saccades after varying delays. The longer it took the monkey to execute the saccade, the smaller the magnitude of the CS signal, tentatively linked to trial onset, got (Fig 7E–7G). This dependency may suggest a role of the trial onset–related CS signal in predicting the upcoming target jump. The rationale is that reaction times should be the shorter the better the ability to predict the timing of the preceding target jump is. A role of the CS in predicting the time of target-directed saccades is in accordance with previous work on eyelid conditioning in mice [21], in which CS bursts elicited by the conditioned stimulus predict the time of occurrence of the unconditioned stimulus.

### Multiplexing of different streams of information by complex spikes

In this study, we could demonstrate that individual PCs are influenced by different types of task-related information that mark distinct elements in the sequence of events relevant to the behavior at stake (Fig 8A and 8B). These different streams of information are multiplexed by individual climbing fibers in a manner that ensures the required precise alignment of information and behavioral events. We further investigated if there was a relationship between the sets of parameters encoded by individual PCs. To this end, we performed a cross-correlation analysis (Fig 8C) and found only a moderate correlation between corrective saccade direction and error magnitude and direction, which is not unexpected since corrective saccades are made in the direction of errors.

What might the purpose of multiplexing be? A speculative answer might be provided by a revision of the classical MAI theory of cerebellar learning [1–3] in which the role of the climbing fiber system is confined to providing information on performance errors driving learning. In our case, the relevant error signal, conveyed via the climbing fiber system, is the retinal error resulting from natural variability in saccade endpoints. Undeniably, information on error feedback is extremely important for optimal motor control, although this information arrives late. But why might the impact of error-related information need this prelude of information on saccade metric? A possible answer is provided by considering that a given error will have very different consequences depending on the features of the saccade that prompted it. Certainly, a 2-deg error resulting from a small amplitude saccade will require adjustments that differ from those of the same error as a consequence of a much-larger saccade. Hence, we suggest that the saccade-related information provided by PC CSs may be an efference copy, serving as a reference signal. This reference signal may ensure a more targeted usage of the subsequent information on error for the benefit of future saccades. Useful information conveyed by CSs may become available even before a primary saccade. This was demonstrated by Junker and colleagues [20] who showed that information on past errors leaves a signature on CS firing that determines the metrics of the upcoming saccade. As said earlier, the idea that the climbing fiber system is able to tap different sources of information useful for optimal behavioral control is well in line with recent thinking about the underpinnings of eyelid conditioning [21]. Actually, not only the CS activity seems to multiplex information but also the PC SS signal. This was recently suggested by Hong and colleagues [60] who demonstrated that specific aspects of eye movement are represented by the SS rate code as well as their timing. While the infrequent pause initiating SSs provide a temporally reliable signal for saccade onset, the frequent, nonpausing SSs provide a firing rate-based linear encoding of saccade kinematics. We speculate that the multiplexed CS code, conveyed via CS firing rate and duration, might provide PCs with a rich spectrum of behaviorally relevant information to choose from that in turn might allow the SSs to influence movement timing and kinematics, also in a multiplexed manner.

But how could different streams of information be merged in the first place, especially when each adult PC receives input from a single climbing fiber originating from an individual neuron in the contralateral inferior olive [61]? The idea that optimal control may rely on dynamic encoding of more than one source of information by CSs [22,62], always relying on the most promising ones, is a concept that has clear implications for the functional architecture of the olivary system. Clearly, this merging cannot be a simple consequence of the convergence of different climbing fibers each reflecting a different input to the olive. Rather, these different streams of information must converge at the level of individual neurons of the inferior olive. How could this be possible given that limited anatomical data suggests that different olivary afferents contact different parts of an olivary nucleus [63]? As a matter of fact, the needed exchange of anatomically disparate information in the inferior olive might be mediated by the well-known gap junction system interconnecting individual neurons in the inferior olive [15,16,64,65]. It has been recently demonstrated that individual inferior olive neurons, rather than being randomly connected, may appear as distinct clusters, each cluster comprising a network of up to 20 closely connected neurons [66]. Hence, under the influence of neighboring clusters receiving distinct inputs, the gap junction–mediated crosstalk between individual neurons, or groups of neurons, may allow a particular climbing fiber to carry more than one type of information. Given the well-established cerebellar feedback-driven GABAergic influence of nucleo-olivary projections on gap junctions [64], we speculate that the cerebellar cortex might play a role in the prioritization of specific inputs. In conclusion, the availability of multiplexed information at the level of PCs pertinent for learning might be based on the flexible control of olivary gap junctions.

## Supporting information

S1 Fig Influence of the repetitive saccade task on saccade vigor. **(A)** Behavioral results from a single experimental session showing a gradual decline in saccade peak velocity (top panels), paralleled by increasing duration (middle panels) to maintain saccade amplitudes (bottom panels) in case of both CF (right column) and CP (left column) saccades. Solid green curves show the trend using a second order polynomial fit. Dark and bright shaded regions in blue and red represent the early and late 30 trials, respectively. **(B)** Horizontal eye positions (upper panel) and velocity profiles of CF (red) and CP (blue) of early and late 30 trials from the same session as shown in A. The effect of fatigue in the later part of the trials in the CF (light red) and CP (light blue) saccades can be seen as the “widening” (i.e., longer duration) of the inverted bell-shaped velocity profiles of saccades in conjunction with a reduction of the peak velocities. **(C)** Range of CF (red) and CP (blue) saccade endpoints scattered around the target location and fixation point (dashed horizontal line). Dots represent the median values for individual sessions. **(D)** Distribution of CF (mean = 14.91 deg) and CP (mean = 14.85 deg) saccade endpoints from all sessions reveal that both types of saccades under- and overshot the target causing retinal errors in both directions. Underlying data available from the Dryad Digital Repository: [https://doi.org/10.5061/dryad.d51c5b03m]. CF, centrifugal; CP, centripetal.
(EPS)

S2 Fig Relationship of CS response to saccade end, CS responses to CF and CP saccades, and a comparison of CF and CP saccades made in the same direction in an exemplary PC. **(A)** CS population response aligned to primary saccade offset. Note how the trough, which marks the beginning of large modulation (gray shaded region), occurs at the time of saccade end (dotted gray line). **(B)** A schematic illustration demonstrating the estimation of modulation onset (trough times). The solid green curve represents the second order polynomial fit to data during −200 to 0 ms from saccade onset (light green shaded region). Solid red line represents the linear fit to data, 45 ms from the time of peak firing toward saccade onset. The intersection of these 2 curves estimates the time of the trough. **(C)** The dotted green line demonstrates the linear relationship (R-sq = 0.86, *p* = 0.007) between saccade duration and modulation onset (trough time, bootstrapped mean ± SEM). **(D)** CS population response (mean ± SEM) aligned to the onset of primary saccades made in the CP (green) and CF direction (purple). **(E, F)** Averaged CS responses (± SEM) of an exemplary PC for CF saccades made in left and right directions, recorded in separate sessions. A comparison of left CF (blue) and left CP (gray) saccades is shown in E, and the comparison of right CP (orange) and right CF (brown) saccades is shown in F. In both cases, the peak CS firing during the *“early postsaccadic period”* (gray shaded region) did not differ if the leftward or rightward saccades started from different locations (E, peak firing rate ± SEM: left CF = 3.3 ± 0.76 spikes/s, left CP = 3.41 ± 0.91 spikes/s, Wilcoxon signed-rank test, *p* = 0.61, z = 0.5; F, peak firing rate ± SEM: right CF = 1.82 ± 0.69 spikes/s, right CP = 1.57 ± 0.47 spikes/s. Wilcoxon signed-rank test, *p* = 0.95, z = 0.06). Underlying data available from the Dryad Digital Repository: [https://doi.org/10.5061/dryad.d51c5b03m]. CF, centrifugal; CP, centripetal; CS, complex spike; PC, Purkinje cell.
(EPS)

S3 Fig Analysis of encoding of primary saccade metrics, separately for CSs in PD_*ps*_ and PD_*ps*_+180°. **(A)** CS population response (mean ± SEM) aligned to the onset of primary saccades sorted by different amplitudes in PD_*ps*_. **(B)** The relationship between saccade amplitudes and peak firing rate of CSs in PD_*ps*_ is demonstrated with the help of a linear regression (R-sq = 0.97, *p* < 0.001). The dashed gray line represents the regression fit. Error bars represent the SEM around the mean. **(C, D)** Same as A and B, but for PD_*ps*_+180° (R-sq = 0.96, *p* < 0.001). **(E**, **G)** CS population response (mean ± SEM) to saccades sorted by duration in PD_*ps*_ and PD_*ps*_+180°, respectively. **(F, H)** The relationship between the timing of the peak response and saccade duration in PD_*ps*_ (R-sq = 0.95, *p* < 0.001) and PD_*ps*_+180° (R-sq = 0.82, *p* = 0.013), respectively. Error bars represent the confidence intervals around the bootstrapped mean. **(I, K)** CS population response (mean ± SEM) to saccades sorted by peak velocity in PD_*ps*_ and PD_*ps*_+180°, respectively. **(J, L)** The relationship between the peak firing rate and saccade peak velocity in PD_*ps*_ (R-sq = 0.04, *p* = 0.78) and PD_*ps*_+180° (R-sq = 0.49, *p* = 0.12), respectively. Error bars represent the SEM around the mean. Underlying data available from the Dryad Digital Repository: [https://doi.org/10.5061/dryad.d51c5b03m]. CS, complex spike.
(EPS)

S4 Fig Analysis of encoding of corrective saccade metrics, separately for CSs in PD_*corr*_ and PD_*corr*_+180°. **(A, B)** CS population response aligned to the onset of corrective saccades, sorted by amplitude (insets), made in PD_*corr*_ and PD_*corr*_+180°, respectively. **(C, D)** The relationship between CS peak firing rate and corrective saccade amplitude in PD_*corr*_ and PD_*corr*_+180°, respectively. The linear regression fit is given by the dotted green line (PD_*corr*_: R-sq = 0.97, *p* = 0.02; PD_corr_+180°: R-sq = 0.78, *p* = 0.12). Error bars represent the mean ± SEM. **(E, F)** CS population response aligned to the onset of corrective saccades, sorted by duration (insets), made in PD_*corr*_ and PD_*corr*_+180°, respectively. **(G)** The relationship between the peak timing of the CS response in PD_*corr*_ (R-sq = 0.8, *p* = 0.29). **(H)** The relationship between saccade duration and modulation onset in PD_*corr*_ (R-sq = 0.58, *p* = 0.45). **(I, J)** Same as G and H, except in PD_*corr*_+180° (I, R-sq = 0.95, *p* = 0.14, and J, R-sq = 0.99, *p* = 0.06). The dotted green lines represent the fits derived from linear regressions. Error bars represent the bootstrapped mean ± confidence intervals. Underlying data available from the Dryad Digital Repository: [https://doi.org/10.5061/dryad.d51c5b03m]. CS, complex spike.
(EPS)

S5 Fig Primary saccades made in the same direction but resulting in opposite errors evoke similar CS responses. **(A)** CS response of an exemplary PC to CF (PD_*ps*_+180°, in blue) and CP (PD_*ps*_, in red) primary saccades (left column). **(B, C)** When saccades are sorted according to the direction of errors (see the schematic diagram on the top of panels B and C), the overall CS response for saccades with leftward error (solid gray trace, lower middle panel) looks very similar to the response for all saccades with rightward errors (solid gray trace, lower right panel). Note that regardless of the direction of the resulting errors (compare dashed red arrows in upper panels B and C), the CSs maintain their specificity for the rightward primary saccades (compare red traces in lower panels B and C). Middle row: raster plots showing the response of an exemplary PC to CF (SS: blue dots and CS: blue circles, respectively) and CP (SS: red dots and CS: red circles, respectively) saccades. Bottom row: average (± SEM) CS firing response of all trials in each condition. Data are aligned to saccade onset. Underlying data available from the Dryad Digital Repository: [https://doi.org/10.5061/dryad.d51c5b03m]. CP, centripetal; CS, complex spike; PC, Purkinje cell; SS, simple spike.
(EPS)

S6 Fig The influence of primary saccade amplitude in PD_*ps*_ and PD_*ps*_+180° with comparable error sizes. **(A, B)** Comparison of all large (red) and small (blue) amplitude saccades in PD_*ps*_ (Wilcoxon signed-rank test, early: *p* < 0.001, late: *p* = 0.55) and PD_*ps*_+180° (Wilcoxon signed-rank test, early: *p* < 0.001, late: *p* = 0.39), respectively. Light gray horizontal lines represent the 0–100-ms period from primary saccade offset (i.e., *“early postsaccadic period”*) and the dark gray horizontal lines denote the 100–250-ms period from primary saccade offset (i.e., later part of *“late postsaccadic period”*), chosen in order to avoid the overlap between these 2 periods. Mean CS firing calculated during these periods was used for statistical comparison. All significant differences (*p* < 0.001) are marked by asterisks. All data are aligned to primary saccade offset (vertical dashed gray line). Underlying data available from the Dryad Digital Repository: [https://doi.org/10.5061/dryad.d51c5b03m]. CS, complex spike.
(EPS)

S7 Fig A multiple regression analysis testing the influence of primary saccades and errors on CS activity during nonoverlapping postsaccadic periods. **(A)** Multiple regression analysis demonstrating the influence of saccade amplitude as well as error direction and magnitude, the latter ranging from PD_*error*_ to PD_error_+180°, on the probability of CS firing (indicated by color bars) during the *“early postsaccadic period*.*”* Given that errors of different sizes could occur in either direction (PD_*error*_ or PD_*error*_+180°) depending on the amplitude and direction (CF or CP) of primary saccades, regression coefficients were obtained for each PC using the equation, Y_i_ = *l*_*i*_ A_*i*_ + *m*_*i*_E_*i*_ + c_i_, where A is saccade amplitude, E is error, Y is the number of CSs that fired during the period of interest, *l* and *m* are slopes of regression, c is the intercept, and subscript *i* is the PC ID. The population mean of slopes, represented as the gradient of colors (mean l = 0.0096; mean m = 0.012) and intercept (mean c = −0.96) allowed us to predict CS firing probabilities for individual values of saccade amplitudes and errors. **(B)** Same as A, except that now data from the *“late postsaccadic period”* was used (mean l = 0.008, mean m = 0.029, and mean c = −0.005). In order to avoid overlap between these periods, a narrow period of 40–80 ms capturing the peak response during the *“early postsaccadic period”* and a period of 100–250 ms from *“late postsaccadic period”* was chosen. Note how the color changes as the error vector changes from −2 deg (i.e., 2 deg in PD_*error*_+180°) to +2 deg (PD_error_) and also with saccade amplitude (13 to 17 deg) in A. However, in B, this color change is mostly observed for errors. Underlying data available from the Dryad Digital Repository: [https://doi.org/10.5061/dryad.d51c5b03m]. CF, centrifugal; CP, centripetal; CS, complex spike; PC, Purkinje cell.
(EPS)

S8 Fig Complementary changes in CS duration and firing rate. **(A)** Population percentage change in CS duration (mean ± SEM) aligned to primary saccade onset. The period of decrease (−55 to 87 ms from saccade onset, shaded region in light red) and increase (94 to 152 ms from saccade onset, light blue shaded region) in CS duration were estimated whenever the response crossed the threshold. **(B)** Population firing response of CSs (mean ± SEM) aligned to primary saccade onset. The period of decrease (−76 to 84 ms from saccade onset, shaded region in light red) and increase (92 to 134 ms from saccade onset, light blue shaded region) in CS firing rate were estimated whenever the response crossed the threshold. Solid horizontal lines in A and B represent the mean values during the baseline period (−200 to −100 ms from saccade onset), and horizontal dashed lines represent the threshold (3×SD around the mean). Note how the regions of decrease and increase in CS duration and firing rate, respectively, largely overlap. Underlying data available from the Dryad Digital Repository: [https://doi.org/10.5061/dryad.d51c5b03m]. CS, complex spike.
(EPS)

S1 Table Summary of all PCs recorded from each monkey for CF saccades in left, right, and both directions. The denominator in the first 3 columns corresponds to the number of PCs recorded, and the numerator corresponds to the number of PCs included in the analysis. In the fourth column, the denominator corresponds to the number of cells recorded in a particular direction, and the numerator corresponds to the subset of those cells recorded in the opposite direction. For example, out of 52 PCs recorded (and also analyzed) in the right CF direction for monkey E, 4 PCs were also tested in the opposite left CF direction. CF, centrifugal; PC, Purkinje cell.
(XLSX)

## Abbreviations

CF: centrifugal
CP: centripetal
CS: complex spike
MAI: Marr–Albus–Ito
OMV: oculomotor vermis
PC: Purkinje cell
SS: simple spike
